# Exploring ethnicity dynamics in Wales: a longitudinal population-scale linked data study and development of a harmonised ethnicity spine

**DOI:** 10.1136/bmjopen-2023-077675

**Published:** 2024-08-02

**Authors:** Ashley Akbari, Fatemeh Torabi, Stuart Bedston, Emily Lowthian, Hoda Abbasizanjani, Richard Fry, Jane Lyons, Rhiannon K Owen, Kamlesh Khunti, Ronan Lyons

**Affiliations:** 1 Population Data Science, Swansea University Medical School, Swansea, UK; 2 Diabetes Research Centre, University of Leicester, Leicester, UK

**Keywords:** PUBLIC HEALTH, EPIDEMIOLOGY, Health policy

## Abstract

**Abstract:**

**Objective:**

This study aims to create a national ethnicity spine based on all available ethnicity records in linkable anonymised electronic health record and administrative data sources.

**Design:**

A longitudinal study using anonymised individual-level population-scale ethnicity data from 26 data sources available within the Secure Anonymised Information Linkage Databank.

**Setting:**

The national ethnicity spine is created based on longitudinal national data for the population of Wales-UK over 22 years (between 2000 and 2021).

**Procedure and participants:**

A total of 46 million ethnicity records for 4 297 694 individuals have been extracted, harmonised, deduplicated and made available within a longitudinal research ready data asset.

**Outcome measures:**

(1) Comparing the distribution of ethnicity records over time for four different selection approaches (latest, mode, weighted mode and composite) across age bands, sex, deprivation quintiles, health board and residential location and (2) distribution and completeness of records against the ONS census 2011.

**Results:**

The distribution of the dominant group (white) is minimally affected based on the four different selection approaches. Across all other ethnic group categorisations, the mixed group was most susceptible to variation in distribution depending on the selection approach used and varied from a 0.6% prevalence across the latest and mode approach to a 1.1% prevalence for the weighted mode, compared with the 3.1% prevalence for the composite approach. Substantial alignment was observed with ONS 2011 census with the Latest group method (kappa=0.68, 95% CI (0.67 to 0.71)) across all subgroups. The record completeness rate was over 95% in 2021.

**Conclusion:**

In conclusion, our development of the population-scale ethnicity spine provides robust ethnicity measures for healthcare research in Wales and a template which can easily be deployed in other trusted research environments in the UK and beyond.

**STRENGTHS AND LIMITATIONS OF THIS STUDY**

- A comprehensive approach for harmonising ethnicity records from population-scale linked data.
- Creating an anonymised longitudinal, individual-level linked ethnicity spine for Wales.
- Enabling investigation and evaluation of health inequalities related to ethnic groups in Wales.
- Providing a reproducible maintainable research ready data asset.
- As this is a data-driven approach, the methodology is limited to the reported ethnicity and diversity of the data and population.

## Introduction

Describing disease dynamics across and within different subgroups of a population and addressing ethnic health disparities in the context of population health and biomedical research is of high importance due to its potential to depict areas of health inequalities and inform decisions towards a fair distribution of services.^1^ Race and ethnicity are often categorised as nonmodifiable social determinants, however, due to the sensitive nature of these data, both self-reported and routinely collected data on ethnicity/race have shown evident changes in reporting and completeness over time.^2^,^5^ These changes, alongside the sensitive nature of the ethnicity data, contribute majorly to available data on ethnicity.^6 7^ Improving existing ethnicity measures and strategies for enriching these data for research has become an urgent public health priority, specifically postobservation of disparities during the COVID-19 pandemic.^8 9^ This improvement can be made by harnessing the available ethnicity information in routinely collected health data. While reports of COVID-19 outcomes demonstrated severe outcomes for minority ethnic groups,^10^,^14^ ethnicity is not always consistently recorded, nor are they available across all data sources and for the entire population. The availability of ethnicity data directly affects our ability to describe differences in a variety of health and social outcomes.^8^ Understanding the dynamics of COVID-19 and its outcomes has further revealed the enduring disparities experienced by ethnic minority groups and the need for improvements in the readiness of these data.^15 16^

The importance of research readiness for investigating the effectiveness of interventions across and within different ethnic groups emphasises the value of ethnicity data. The availability of ethnicity data across the UK has been documented.^17^ Enabling ethnicity-level characterisation of the population has been highlighted across various disease groups,^18^ in previous pandemics^19^ and among the elderly.^20^ Research into health inequalities across different ethnic groups has continually referenced the diverse manifestation of coded ethnicity in data, including clinical and administrative data sources, and its impact on the researchers’ ability to estimate disparities.^15 21^ While the census provides a current snapshot of the geographical distribution of ethnicity records across the population, the culmination of the census data from 2011 means that embracing the recapturing opportunity through linkage of routinely collected ethnicity information in electronic health record (EHR) data provides a current measure at any given time point. There is current evidence of crude approaches to processing ethnicity data, for example, the label ‘South Asian’ often includes the subpopulation groups, who are often culturally and behaviourally different from each other, for example, Bangladeshi, Pakistani and Indian.^22^ Stakeholder groups have also emphasised that the data collected on ethnicity should be ‘strengthened’ towards enabling the inclusion of all ethnicities in the research.^12^

Data diversity provides a unique opportunity to access recorded ethnicity across multiple data sources and permits the harmonisation of approaches to examine the consistency of research outputs and conclusions, which contributes to an improved understanding of health outcomes in marginalised ethnicities. Here, we aimed to improve harmonised ethnicity data. We assessed the availability, record completeness and distribution of ethnicity records across EHRs and administrative data sources. We compared four different methods for deriving ethnicity from multiple data sources referred to as record selection approaches. The four selection approaches are namely: latest, mode, weighted mode and composite. In addition to selection approaches, we also evaluate the impact of two different ways of categorising ethnicity: from the New and Emerging Respiratory Virus Threats Advisory Group (NERV-TAG) referred to as NER hereafter, and the Office for National Statistics (ONS).^15 16^ NER consists of nine different categories with higher granularity for ‘Asian’ and ‘black’ groups while ONS consists of five categories both are detailed in method section.^15 16^ Our work directly responds to the national need for these data, specifically in the context of health.^8 23^

## Methods

### Study design and data sources

**Figure 1:**
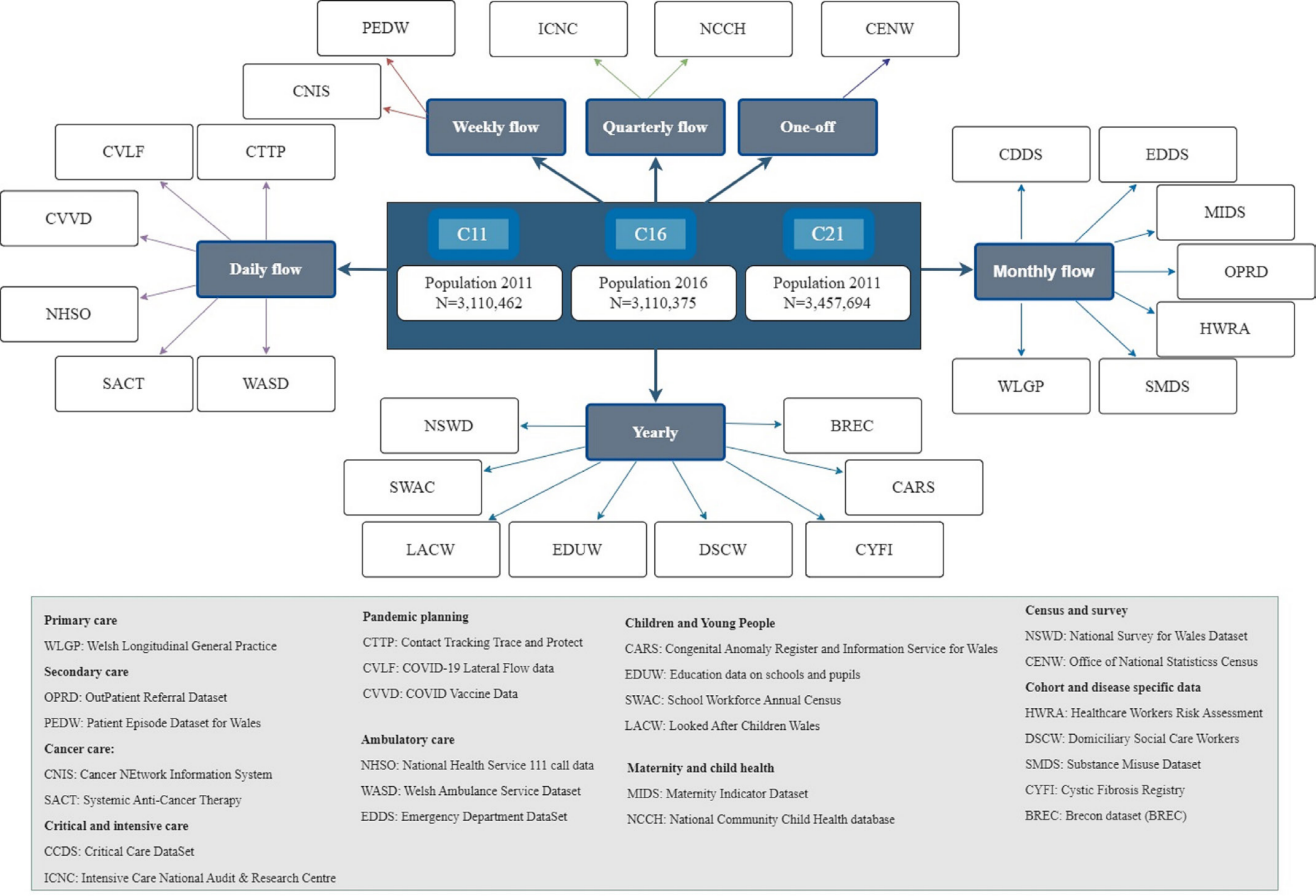
Data sources used to create the harmonised ethnicity spine for Wales (data sources presented based on the frequency of information flow).

All anonymised individual-level, population-scale data sources within the Secure Anonymised Information Linkage Databank trusted research environment (TRE)^24 25^ were assessed for the availability and completeness of ethnicity records, the details on how completeness is quantified is provided in statistical analysis section. We used longitudinal records spanning 22 years, with the earliest records starting from 1 January 2000 and the most complete range of data providing full-year data up to 31 December 2021. Ethnicity records were extracted from 26 EHRs and administrative data sources based on their associated available coverage contributing to the 22 years longitudinal study window. Contributing data sources had various update frequencies ranging from daily flows to quarterly flows (figure 1, online supplemental figure 1 and online supplemental table 1).

The extracted information from linked data sources gets compiled in a longitudinal dataset, in a stepwise manner to create a population-spine ethnicity research ready data asset (RRDA). These steps are (1) record extraction, where all existing ethnicity records for each individual are extracted from the 26 data sources over time and gathered in a longitudinal dataset (see online supplemental table 2) for details on record extraction from each datatable); (2) The record cleaning where any duplicated records or missing values gets cleaned and all values are harmonisation into ethnic groups using two different categorisation methods: NER and ONS^15 16^—we refer to this stage as categorisation hereafter) and finally (3) serialising these records into a single row per individual using four different selection approaches: composite, latest, mode, weighted mode—to assign a single ethnicity to each individual (we refer to this stage as record selection hereafter). All longitudinal ethnicity records are preserved, and changes over time for each individual are accessible, enabling the allocation of an individual-level ethnic grouping at any point in time to any anonymised linkable individual records. Approximately 4% of the Welsh population had two contradicting ethnicity records over time. In the record extraction stage: from each respective data source, we only extract the records that has a reliable linkage quality (ie, a match percentage of 95% quality or higher) and the date of the record occurring within the desired study window for all available longitudinal records. This will enable a reliable linkage process between all sources. Postextraction, all records are organised longitudinally for each data source, and all values are harmonised into two different ethnicity categorisations: consisting of five categorisations: ‘white’, ‘Asian’, ‘black’, ‘mixed’, ‘other’ as proposed by the ONS^16^, and a more granular level of categorisation using nine ethnic group categories: ‘white’, ‘Chinese’, ‘Bangladeshi’,’ Pakistani’,’ Indian’, ‘Black African’, ‘black Caribbean’, ‘mixed’, ‘other’ as proposed by NER.^15^ We investigate the effect of the two categorisation approaches on longitudinal assignment of ethnicity records. Where we found multiple ethnicity recordings for an individual in various sources or over time, all records were ordered sequentially based on the date of recorded ethnicity. Finally, to reshape and process the data into one row per person, in the selection stage, we first select the most recent ethnic group category, which is the common approach in various research projects.^26 27^ This is called the ‘latest’ method and is compared against three different selection approaches for the assignment of a single ethnic group to each individual:

1. Latest: The most recent ethnic group available in any of the available data sources is assigned to an individual as their ethnic group.
2. Mode: The most frequently recorded ethnic group (the mode) is extracted from all available data sources and is assigned to an individual as their ethnic group.
3. Weighted mode: The weighted mode assigns weights to each ethnic group category extracted from all available data sources, based on the inverse proportion of the prevalence reported by the^16^ Census, with the highest frequently recorded ethnic group following weighting being assigned to an individual as their ethnic group.
4. Composite: The ethnicity of those individuals who have declared more than one distinct ethnic group over time is assigned to the Mixed ethnic group (due to the nature of the composite method and no hierarchy or prioritisation given to which ethnic group should be assigned, where different categories are found for an individual over time, the composite method would assign all of the 4% of individuals with contradicting records into the mixed ethnic category).

To create a Common Data Model (CDM) for population-scale data on ethnicity, all available records within each data source are harmonised and categorised into the available ethnic group categories based on clinical and domain expertise (see online supplemental table 3) for the metadata of RRDA tables, postassessment, harmonisation and creation of a CDM for population-scale data on ethnicity, we summarise all this information in our ethnicity spine RRDA, to provide harmonised ethnic group information suitable for data linkage. The RRDA is maintained over time and is available to researchers using the SAIL Databank.

### Statistical analysis

Distribution of ethnicity records for each selection method (latest, mode, weighted mode and composite) and the two categorisation methods (NER and ONS) are compared over time. We also assessed completeness of data over time. Three cohorts consisting of individuals alive and living in Wales in 2011, 2016 (mid-study) and 2021 (end of study) (figure 1) were used to compare record completeness over time from the year the census was completed in 2011. Using the ONS and NER ethnic group categorisations, we compare the distribution of records for the four selection approaches across age bands, sex, deprivation quintiles, geographical health boards and residential areas. Cohen’s kappa coefficient was calculated to assess the inter-rater reliability between the latest ethnicity approach and census records for the 2011 population.^28^ We report the inter-rater coefficients for those with known ethnicity status in both the census and the latest approach. Where 0 is defined as no agreement between the two sources, and 1 is the maximal agreement of records across the two sources. Age is calculated based on the week of birth of individuals on 1 January for each population or later if born afterwards. Week of birth, sex, health board and residential information of all individuals are sourced from Welsh Demographic Dataset^29^ and in the COVID-19 e-cohort known as C20.^30^

### Patient and public involvement

This project is undertaken under a proposal submitted to the independent Information Governance Review Panel (IGRP), including members of the public (IGRP Project: 0911). Two public members contributed to the project’s approval as part of the IGRP panel.

## Results

### Cohort curation

A total of 46 million ethnicity records were recorded between 2000 and 2021 across the 26 EHRs and administrative data sources for 4 297 694 individuals (table 1). We summarised the data for the population at three inception dates for all individuals who are alive and residing in Wales at that date: 1 January 2011 (C11) for 3 110 462 individuals, 1 January 2016 (C16) for 3 110 375 and 1 January 2021 (C21) for 3 457 697. Each cohort contains records of individuals alive and residing in Wales on 1 January of each year.

**Table 1:** Data source range, number of individual and total ethnicity records in each contributing data source (breakdown of the ethnic group is presented using ONS categorisation)

Data source	(Start date, end date)	Number of people	Number of ethnicity records	Asian	Black	Mixed	Other	White
BREC	(1 January 2000, 19 April 2021)	3787	3787	65	36	76	20	3590
CARS	(3 January 2000, 31 December 2018)	17 581	19 065	336	189	167	154	16 735
CCDS	(1 April 2006, 31 December 2021)	90 303	108 100	507	151	107	95	89 493
CENW	(27 March 2011, 27 March 2011)	2 663 411	2 663 726	55 865	13 207	26 001	10 150	2 558 451
CNIS	(1 January 2000, 31 December 2021)	43 998	127 854	119	37	86	103	43 653
CTTP	(1 June 2020, 1 December 2021)	364 093	444 833	8526	2230	6868	3689	342 828
CVLF	(16 October 2019, 31 December 2021)	1 880 259	11 225 043	55 307	18 556	30 487	14 984	1 779 094
CVVD	(3 March 2019, 31 December 2021)	1 579 429	4 164 721	39 117	10 196	12 940	20 494	1 496 818
CYFI	(1 January 2000, 31 December 2020)	11 188	136 953	343	49	65	132	10 655
DSCW	(1 June 2020, 1 June 2020)	24 693	24 693	214	283	244	127	23 825
EDDS	(5 April 2008, 31 December 2021)	762 737	2 706 047	48 669	2595	3909	3361	719 605
EDUW	(1 January 2003, 1 January 2020)	1 124 234	8 121 162	26 650	10 934	34 707	14 971	1 053 707
HWRA	(31 January 2020, 31 December 2021)	111 384	2 139 617	5617	1369	1322	134	103 173
ICNC	(1 April 2009, 30 June 2021)	100 328	121 401	864	292	210	248	98 802
LACW	(31 March 2003, 31 March 2021)	12 451	61 943	303	249	497	264	11 408
MIDS	(31 March 2014, 31 December 2021)	127 681	314 224	5697	2222	2163	2376	115 737
NCCH	(1 January 2000, 8 October 2021)	609 453	126,6931	19 686	7904	16 043	9879	557 264
NHSO	(1 February 2020, 31 December 2021)	14 852	26 273	103	26	87	31	14 605
NSWD	(31 March 2014, 31 March 2019)	40 100	40 261	448	131	190	132	39 200
OPRD	(10 January 2000, 31 December 2021)	822 337	3 271 792	11 039	4470	4463	4064	800 292
PEDW	(1 January 2000, 31 December 2021)	1 194 215	5 335 310	15 060	12 120	6698	9596	1 159 297
SACT	15 January 2001, 31 December 2021)	Masked	18 690	35	<10	34	31	13 456
SMDS	(1 January 2000, 31 December 2021)	82 167	217 551	574	434	713	154	80 605
SWAC	(1 September 2019, 1 September 2020)	49 016	90 273	311	103	375	116	48 144
WASD	(31 August 2015, 31 December 2021)	342 169	592 144	3173	546	4795	274	338 075
WLGP	(1 January 2000, 31 December 2021)	1 834 740	2 825 204	95 156	27 663	23 720	48 192	1 666 779

BRECBrecon datasetCARSCongenital Anomaly Register and Information Service for WalesCCDSCritical Care DataSetCENWONS Census 2011CNISCancer Network Information SystemCTTPContact Tracking Trace and ProtectCVLFCOVID-19 Lateral Flow dataCVVDCOVID Vaccine dataCYFICystic Fibrosis RegistryDSCWDomiciliary Social Care WorkersEDDSEmergency Department Data SetEDUWEducation data on schools and pupilsHWRAHealthcare Workers Risk Assessment dataICNCIntensive Care National Audit and Research Centre dataLACWLooked After Children Wales dataMIDSMaternity Indicator Data SetNCCHNational Community Child Health databaseNHSONational Health service 111 call dataNSWDNational Survey for Wales DatasetONSOffice for National Statistics OPRDOutPatient Referral DatasetPEDWPatient Episode Databasefor WalesSACTSystemic Anti-Cancer TherapySMDSSubstance Misuse Data SetSWACSchool Workforce Annual Census dataWASDWelsh Ambulance Service DatasetWLGPWelsh Longitudinal General Practice

### Evaluation of different selection approaches for ethnic group categorisation

A comparison of four selection approaches (latest, mode, weighted mode and composite) showed that the distribution of C21 records varied based on approach: for the mixed ethnic group using the latest and mode method resulted in 1.3% of the population being assigned to mixed group while using weighted mode resulted in 2.0% assignment, this has raised to 6.0% in the composite approach, in which anyone with more than one distinct ethnic group category is assigned to the mixed group (ONS mixed latest=1.3%, ONS mixed mode=1.3%, ONS mixed weighted mode=2.0%, ONS mixed composite=6.0%) similar patterns were observed for the NER categorisation (NER mixed latest=1.3%, NER mixed mode=1.3%, NER mixed weighted mode=1.9%, NER mixed composite=6.3%) (table 2). Breaking down the records for each approach by sex and age for the two ONS and NER categorisations showed that the white ethnic group was recorded as the highest proportion, and its distribution was minimally affected by the approach used (female white ONS latest=44%, female white ONS mode=44%, female white ONS weighted mode=43%, female white ONS composite=43% and female white NER latest=44%, female white NER mode=44%, female white NER weighted mode=43%, female white NER composite=43%). Across all the minority ethnic groups, the mixed group was the group for which the record distribution varied from the latest and mode compared with the composite approach (female mixed ONS latest=0.6%, female mixed ONS mode=0.6%, female mixed ONS weighted mode=1.1%, female mixed ONS composite=3.1% and female mixed NER latest=0.6%, female mixed NER mode=0.6%, female mixed NER weighted mode=1.0%, female mixed NER composite=3.2%) (online supplemental figures 2 and 3). While for the rest of the ethnic groups, the distribution of records across age bands and sex was similar for four approaches across both ONS and NER categorisations, we illustrated these across all age bands in online supplemental figures 2 and 3 (online supplemental table 4 and 5 for distribution of records over each age band and selection method). We observed an improved categorisation achieved by the latest method followed by the mode selection method in nine NER categorisation.

**Table 2:** Characteristics of the cohort based on ONS and NER categorisation

Characteristics	Latest ethnic groupN=3 457 694N (%)		Mode ethnic groupN=3 457 694N (%)		Weighted mod ethnic groupN=3 457 694N (%)		Composite ethnic groupN=**3** 457 694N (%)	
Ethnic group (ONS)								
White	3 080 277	(89.0)	3 093 752	(89.0)	2 989 648	(86.0)	2 987 245	(86.0)
Asian	110 809	(3.2)	100 502	(2.9)	133 258	(3.9)	72 564	(2.1)
Black	30 880	(0.9)	30 817	(0.9)	41 559	(1.2)	19 036	(0.6)
Mixed	43 672	(1.3)	43 422	(1.3)	70 880	(2.0)	207 627	(6.0)
Other	38 724	(1.1)	35 869	(1.0)	69 017	(2.0)	17 890	(0.5)
Unknown	153 332	(4.4)	153 332	(4.4)	153 332	(4.4)	153 332	(4.4)
Ethnic group (NER)								
White	3 080 277	(89.0)	3 093 817	(89.0)	2 987 420	(86.0)	2 987 245	(86.0)
Bangladeshi	25 780	(0.7)	16 243	(0.5)	55 675	(1.6)	9204	(0.3)
Chinese	17 970	(0.5)	17 851	(0.5)	20 302	(0.6)	13 703	(0.4)
Indian	25 058	(0.7)	25 190	(0.7)	27 078	(0.8)	17 075	(0.5)
Pakistani	17 035	(0.5)	17 043	(0.5)	20 287	(0.6)	11 030	(0.3)
Black African	21 578	(0.6)	22 003	(0.6)	32 671	(0.9)	9904	(0.3)
Black Caribbean	4730	(0.1)	4083	(0.1)	7145	(0.2)	1135	(<0.1)
Mixed	43 672	(1.3)	43 572	(1.3)	66 895	(1.9)	217 059	(6.3)
Other	68 262	(2.0)	64 560	(1.9)	86 889	(2.5)	38 007	(1.1)
Unknown	153 332	(4.4)	153 332	(4.4)	153 332	(4.4)	153 332	(4.4)

NERNew and Emerging Respiratory Virus Threats Advisory Group (NERV-TAG) referred to as NERONSOffice for National Statistics

**Figure 2:**
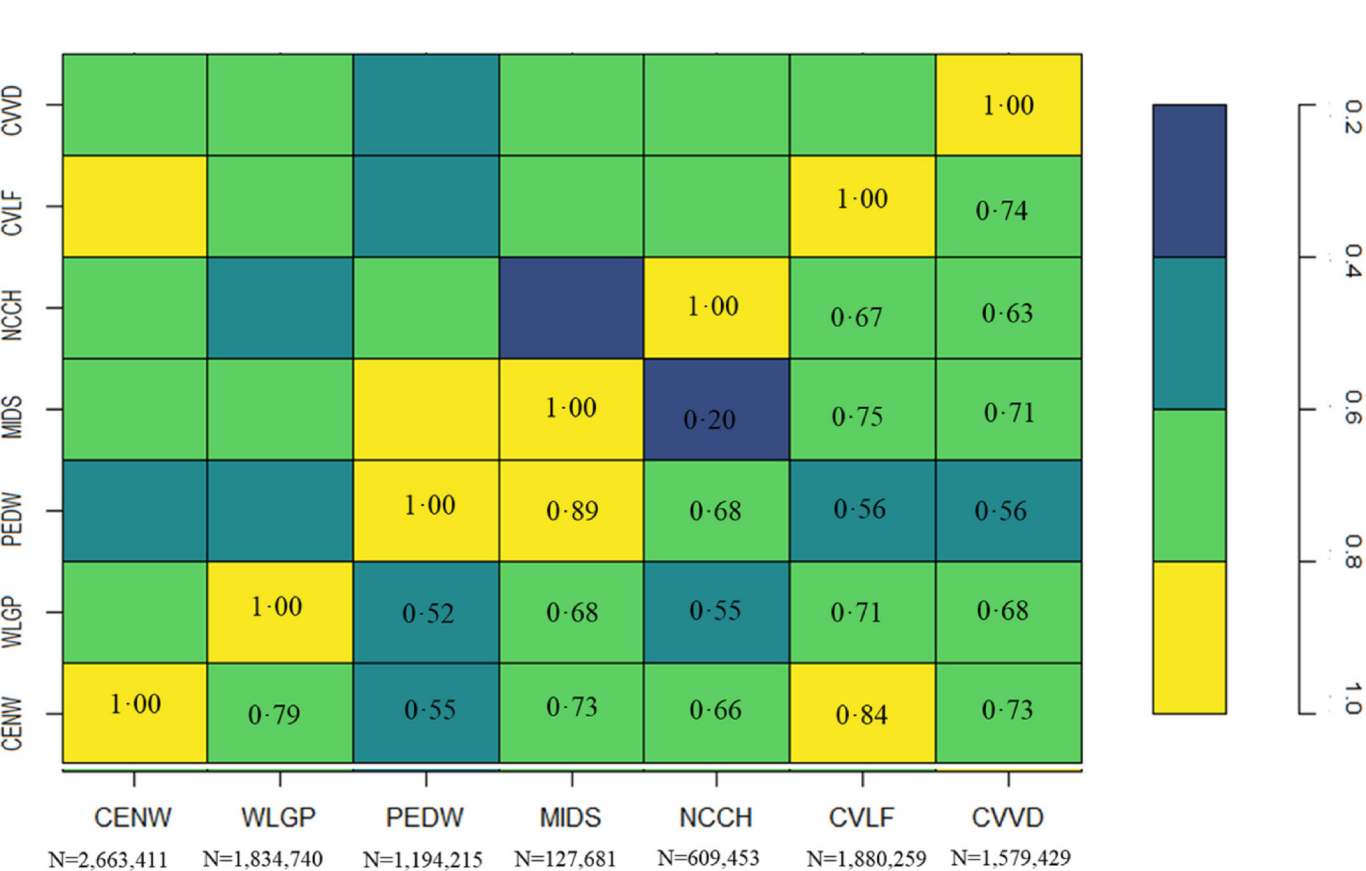
Inter-rater coefficients between existing ethnic groups in Census 2011 (CENW), Welsh Longitudinal General Practice (WLGP), Patient Episode Dataset for Wales (PEDW), Maternity Indicator DataSet (MIDS), National Community Child Health data (NCCH), COVID-19 Lateral Flow data (CVLF) and COVID Vaccine Data (CVVD).

In 2011, census data provided the most complete distribution of ethnicity records, where 2 546 403 (82%) of the C11 population had a recorded ethnicity in the census, leaving 18% of the population without an available ethnic group, known as the unknown ethnic group across both ONS and NER categorisation. The contribution of EHR over time increases from 10% in 2011 to 31% in 2021, resulting in a 13.6% reduction of missingness of an available ethnic group over time for both ONS and NER categorisation with the latest method. This is in the background of changes in the population, including births and moving in and out of Wales (table 3 and online supplemental figure 4). A comparison of individual-level category assignment between those who had a record both in the ethnicity spine and in the census (eliminating 1 663 775 individuals who had an unknown ethnicity either in the census or in the C11 cohort) showed substantial agreement between the census and ethnicity spine RRDA (Cohen’s kappa=0.68, 95% CI (0.67 to 0.71))). Post-2011, record completeness improved by 2.8% from 2016 to 2021, with an agreement level of 0.46% across the 2 years (Cohen’s kappa=0.46, 95% CI (0.44 to 0.49)) (see figure 2 and online supplemental figure 5) for Cohen’s kappa matrix).

**Table 3:** Record comparison for ONS categorisation between the census and the latest method approaches

Characteristics	Census ethnic group 2011 N=3 110 462	Latest ethnic group 2011 N=3 110 462	Latest ethnic group 2016 N=3 110 375	Latest ethnic group 2021 N=3 457 694
Ethnic group source				
Census	2 546 403 (81.9%)	2 546 403 (81.9%)	2 331 911 (75.0%)	2 243 846 (64.9%)
EHR	---	313 211 (10.1%)	554 179 (17.8%)	1 060 516 (30.7%)
Unknown	564 059 (18.1%)	250 848 (8.1%)	224 285 (7.2%)	153 332 (4.4%)
Ethnic group (ONS)				
White	2 451 348 (78.8%)	1 671 303 (53.7%)	2 747 991 (88.3%)	3 080 277 (89.1%)
Asian	50 054 (1.6%)	40 045 (1.3%)	64 997 (2.1%)	110 809 (3.2%)
Black	11 823 (0.4%)	13 730 (0.4%)	19 491 (0.6%)	30 880 (0.9%)
Mixed	24 313 (0.8%)	19 352 (0.6%)	32 777 (1.1%)	43 672 (1.3%)
Other	8865 (0.3%)	15 468 (0.5%)	20 834 (0.7%)	38 724 (1.1%)
Unknown	564 059 (18.1%)	1 350 564 (43.4%)	224 285 (7.2%)	153 332 (4.4%)
Ethnic group (NER)				
White	2 451 348 (78.8%)	1 671 303 (53.7%)	2 747 991 (88.3%)	3 080 277 (89.1%)
Indian	12 445 (0.4%)	10 588 (0.3%)	15 337 (0.5%)	25 058 (0.7%)
Chinese	7928 (0.3%)	6262 (0.2%)	10 439 (0.3%)	17 970 (0.5%)
Bangladeshi	8630 (0.3%)	7952 (0.3%)	11 454 (0.4%)	25 780 (0.7%)
Pakistani	9836 (0.3%)	6059 (0.2%)	12 169 (0.4%)	17 035 (0.5%)
Black African	2441 (0.1%)	9733 (0.3%)	11 158 (0.4%)	21 578 (0.6%)
Black Caribbean	7458 (0.2%)	1689 (0.1%)	4920 (0.2%)	4730 (0.1%)
Mixed	24 313 (0.8%)	19 352 (0.6%)	32 777 (1.1%)	43 672 (1.3%)
Other	22 004 (0.7%)	26 960 (0.9%)	39 845 (1.3%)	68 262 (2.0%)
Unknown	564 059 (18.1%)	1 350 564 (43.4%)	224 285 (7.2%)	153 332 (4.4%)
Age band				
0–19	706 461 (22.7%)	706 461 (22.7%)	686 558 (22.1%)	739 814 (21.4%)
20–39	808 723 (26.0%)	808 723 (26.0%)	788 700 (25.4%)	898 841 (26.0%)
40–59	839 910 (27.0%)	839 910 (27.0%)	840 572 (27.0%)	882 458 (25.5%)
60+	755 368 (24.3%)	755 368 (24.3%)	794 545 (25.5%)	881 107 (25.5%)
Sex				
Male	1 549 319 (49.8%)	1 549 319 (49.8%)	1 551 616 (49.9%)	1 728 015 (50.0%)
Female	1 561 143 (50.2%)	1 561 143 (50.2%)	1 558 759 (50.1%)	1 729 679 (50.0%)
Deprivation quintile				
1-Most deprived	622 879 (20.0%)	622 879 (20.0%)	631 347 (20.3%)	658 913 (19.1%)
2	619 359 (19.9%)	619 359 (19.9%)	619 099 (19.9%)	638 925 (18.5%)
3	627 624 (20.2%)	627 624 (20.2%)	624 789 (20.1%)	643 624 (18.6%)
4	618 525 (19.9%)	618 525 (19.9%)	615 938 (19.8%)	630 614 (18.2%)
5-Least deprived	622 075 (20.0%)	622 075 (20.0%)	619 202 (19.9%)	635 890 (18.4%)
Local health board (HB)				
Aneurin Bevan University HB	588 352 (18.9%)	588 352 (18.9%)	587 737 (18.9%)	605 782 (19.5%)
Betsi Cadwaladr University HB	689 758 (22.2%)	689 758 (22.2%)	683 671 (22.0%)	701 543 (20.3%)
Cardiff and Vale University HB	493 312 (15.9%)	493 312 (15.9%)	505 232 (16.2%)	526 652 (15.2%)
Cwm Taf Morgannwg University HB	444 806 (14.3%)	444 806 (14.3%)	444 300 (14.3%)	459 118 (14.3%)
Hywel Dda University HB	376 346 (12.1%)	376 346 (12.1%)	372 309 (12.0%)	385 174 (12.2%)
Powys Teaching HB	129 363 (4.2%)	129 363 (4.2%)	126 644 (4.1%)	128 461 (3.7%)
Swansea Bay University HB	388 525 (12.5%)	388 525 (12.5%)	390 482 (12.6%)	401 236 (11.6%)
Urban rural classification				
Rural town and fringe	412 275 (13.3%)	412 275 (13.3%)	410 122 (13.2%)	419 900 (13.1%)
Rural town and fringe in a sparse setting	120 097 (3.9%)	120 097 (3.9%)	117 362 (3.8%)	118 480 (3.7%)
Rural village and dispersed	205 555 (6.6%)	205 555 (6.6%)	201 231 (6.5%)	204 993 (6.4%)
Rural village and dispersed in a sparse setting	227 732 (7.3%)	227 732 (7.3%)	221 427 (7.1%)	225 307 (7.0%)
Urban city and town	2 086 855 (67.1%)	2 086 855 (67.1%)	2 102 605 (67.6%)	2 179 684 (68.1%)
Urban city and town in a sparse setting	57 948 (1.9%)	57 948 (1.9%)	57 628 (1.9%)	59 602 (1.9%)

Ethnic group results are presented specifically for ONS and NER categorisation.

EHRelectronic health recordHBhealth boardONSOffice for National Statistics

## Discussion

Our development of the population-scale ethnicity spine provides robust ethnicity measures to healthcare research in Wales and a template which can easily be deployed in other TREs in the UK and beyond. The CDM for ethnicity presented in this study involved categorising and cleaning data and values recorded across 26 longitudinal data sources over 22 years. The combination of the 22 years of ethnicity data and variability in contributing data sources resulted in achieving record completeness of over 95% in 2021 for the entire population of Wales. One main contributing factor to achieving over 95% ethnicity record completeness was the recapturing opportunity available based on multiple data sources and capture points longitudinally, including the more recently acquired COVID-19-related data sources. Two major COVID-19-related data sources: the COVID-19 vaccination (CVVD) and the lateral flow testing data sources, have provided the opportunity of recapturing ethnicity records across the entire Welsh population. When compared with previous years, a 2.8% improvement was achieved in record completeness since 2016. The longitudinal contribution opportunity through multiple data sources provides a capture and recapturing opportunity for the whole population. Our study demonstrates that the latest, mode and weighted mode approaches all provide suitable categorisations for ethnic group data, and the choice of ethnic group methodological approach chosen by any study is down to their respective study design, with the standard choice from current studies across the UK being the latest.

We present a methodology for creating a longitudinal population-scale individual-level linked data ethnicity spine for the population of Wales. Additionally, to assess the robustness of our proposed methodology, we provide a comparison of different categorisation and selection approaches. We further summarised the contribution level of each data source and compared the EHR ethnicity with the^16^ census over time, considering changes in the population dynamics due to births, deaths and migration. Our methodology showed that using the latest method provides a substantial agreement measure of ethnicity in the population when compared directly with the census (Cohen’s kappa=0.68) demonstrating a promising improvement to what has previously been reported on the quality of ethnicity data within the EHRs^2 3 31^ and enabling disaggregation of outcomes by ethnicity.^32^,^34^ Our methodology has demonstrated the benefits of including ethnic group information when understanding clinical outcomes at a population scale. When assessing the useability of the different methods (ie, latest, mode, weighted mode and composite), it is important for users to consider which is most appropriate to their study design based on the available data to their project. The latest method, which is most commonly adopted across research studies, primarily respects an individual’s choice to change their self-declared ethnicity over time and identify how each individual self-identifies at the most recent point in time of their longitudinal records. In comparison, the mode and weighted mode methods allow researchers to take into account variation in reporting between data sources and longitudinally in order to select the most reported ethnic group, which potentially handles for data error and variability to assign the most frequently reported ethnic group over the whole period of their research study period to what ethnic group would be applicable for the whole study period. The composite method does not take into account temporal changes (as with the latest method) or the most declared ethnic group (mode and weighted mode) but instead assigns an ethnic group if unique across all data sources and data coverage available or if two or more different ethnic groups are found then assigns the ethnic group as mixed, without prioritising one record and ethnic group over another. As such, this composite method is limited in its use and applicability and should not be considered a primary method of use.

The continuous recapturing of records across EHRs ensures the under-representation of ethnic minority groups over time is addressed, leading to fewer erroneous results among other studies using this data. This method can further be developed by incorporating self-reported ethnicity measurements with appropriate weightings applied to the data source contributions accounting for self-reported or routinely collected ethnicity information to account for possible effects of record capturing methods and the inherent data quality or reporting bias found from different data sources, systems and settings. We have shown during the COVID-19 pandemic, the availability of various data sources resulted in an opportunity for recapturing ethnicity records. The proposed method in this manuscript has shown usefulness in various research enabling investigations around health inequalities for minority ethnic groups.^2735^,^40^ There are further recent reports on variations in outcomes for ethnic minority groups with documented effects on vaccine hesitancy^41^ as well as postvaccination COVID-19 deaths that were found to be higher among those of Pakistani or Indian background.^42^ Our study provides a population-scale national ethnicity CDM in the form of an RRDA to support ongoing activities for the assessment of health risks, behaviours and outcomes across the less representative subgroups of the population of Wales, enabling the evaluation of potential inequalities due to ethnic groups across the population of Wales. To date, this methodology has enabled national level reporting on vaccination uptake in the general population of Wales^37^ as well as supporting strategic vaccination decisions for subgroups of the population such as healthcare workers.^36^

We explored the effect of categorising people with inconsistent records throughout time into the mixed ethnic group category through the composite method. We have shown that this method skews records for descriptive analysis across the COVID-19 measure. We would like to further outline the limitations of the composite method as demonstrated throughout, with the availability of more data over time contributing to the composite method only by increasing the proportion of records in the mixed group and the decrease in those individuals being assigned to a unique ethnic group category. As such, the composite approach is only appropriate in certain study design circumstances with a fixed point in time, no updates in data coverage and a need to retain the knowledge of individuals that declare to more than one ethnic group over time as a single row per individual variable. We would like to acknowledge that although we had access to a collection of longitudinal data sources within the SAIL Databank, the scope of our work is limited to the quality of records available in each data source. While further improvement in primary data collection and encouragement on accurate reporting of ethnicity is essential, considerations about the changeable nature of this data should be built into data cleaning and research methodology. We have shown in this paper that categorisation of individuals who declared more than one ethnicity over time into a single category can introduce major biases in the true distribution of ethnicity in the population. Additionally, understanding the potential types of data that can be captured that may be mislabelled as ethnicity data, such as race, lineage and country of origin.

Further work is needed to investigate the causes of unknown categories. Unknown in this context included a mixture of values available in multiple data sources, including individuals who may never have been asked their ethnicity, those who chose not to disclose their ethnicity and those who are not found in data sources to be interacting with services. This category includes different types of missingness, such as unknown, null, not declared, not stated and other potential values that may indicate potential variability within and between data sources, especially in potential groups where inequalities are thought to be higher or certain groups who are potentially at greater risk of COVID-19 infection or outcomes such as healthcare providers or social workers. For this study, a pragmatic selection of the data sources available containing ethnicity and the categories based on the requirements to respond to the COVID-19 pandemic was completed. Further work should be done to iterate the available groupings in terms of additional granularity and other potential assignments of available ethnicity values into ethnic group categories based on the different research aims and study designs for future studies that may be interested in specific subpopulations and ethnic groups. This is being discussed via the Health Data Research UK Alliance diversity in data working group for ethnicity, with stakeholders across various disciplines to develop future standards and frameworks that could be standardised in policy and available as harmonised phenotypes.^43^

Our population-level CDM and methodology, using 22 years of longitudinal data in 26 data sources, provides a ground for investigating existing associations and health inequalities at the population level in Wales. It also informs opportunities for the transferability of this methodology across the UK to other data sources and TREs, platforms and systems which hold comparable routine data sources that contain ethnicity information, who can take the learning, harmonisation and implementation of this methodology and replicate it for wider use and further harmonisation and opportunities to implement UK wide inequalities research. The ethnicity spine RRDA is currently available to all COVID-19 researchers within the SAIL Databank to use towards COVID-19 research and evaluations, with further work planned to discuss with data owners around the opportunities to share this implemented RRDA within the SAIL Databank with all researchers in SAIL directly, following appropriate governance and application approvals.

## supplementary material

10.1136/bmjopen-2023-077675 Supplementary file 1

## Data Availability

Data are available on reasonable request.
