# Supplementary material for: Exploring ethnicity dynamics in Wales: a longitudinal population-scale linked data study and development of a harmonised ethnicity spine
Source: BMJ Open. 2024 Aug 2;14(8):e077675. doi: 10.1136/bmjopen-2023-077675 (PMC11733787; doi:10.1136/bmjopen-2023-077675)
Supplement: Supplementary file 1 [file bmjopen-14-8-s001.pdf]

Supplementary materials

Developing a research ready population-scale linked data ethnicity-spine in Wales

Authorship list

Ashley Akbari * [1], Fatemeh Torabi * [1], Stuart Bedston [1], Emily Lowthian [1,3], Hoda Abbasizanjani [1], Rich Fry [1], Jane Lyons [1], Rhiannon Owen [1], Kamlesh Khunti ₸ [2], Ronan A. Lyons ₸ [1]

[1] Population Data Science, Swansea University Medical School, Faculty of Medicine, Health & Life Science, Swansea University, Swansea, Wales, UK
[2] Diabetes Research Centre, University of Leicester, Leicester, LE5 4PW
[3]. Department of Education & Childhood, School of Social Sciences, Swansea University, Swansea, Wales, UK

* Authors have equally contributed
₸ Senior Authors

Contents

Developing a research ready population-scale linked data ethnicity-spine in Wales ... 1
Supplementary Figure 1 ... 3
Supplementary Figure 2 ... 5
Supplementary Figure 3 ... 6
Supplementary Figure 4 ... 7
Supplementary Figure 5 ... 8
Supplementary Table 1 ... 9
Supplementary Table 2 ... 10
- BREC (Brecon data) ... 10
- CARS (Congenital Anomaly Register and Information Services for Wales) ... 11
- CCDS (Critical Care DataSet) ... 12
- CENW (ONS Census 2011) ... 13
- CNIS (CANISC – Cancer Network Information System Cymru) ... 14
- CTTP (COVID-19 Test Trace and Protect) ... 15
- CVLF (COVID-19 Lateral Flow) ... 18
- CVVD (COVID-19 Vaccine Data) ... 19
- CYFI (Cystic Fibrosis Register) ... 20
- DSCW (Domiciliary Social Care Wales) ... 21
- EDDS (Emergency Department DataSet) ... 23
- EDUW (Education data) ... 24
- HWRA (Health Worker Risk Assessment) ... 28
- ICNC (ICNARC Intensive Care National Audit Research Centre) ... 30
- LACW (Looked After Children Wales) ... 31
- MIDS (Maternity Indicators DataSet) ... 32
- NCCH (National Community Child Health data) ... 33
- NHSO (NHS 111) ... 34
- NSWD (National Survey for Wales Data) ... 35
- OPRD (Out Patient Referrals Dataset) ... 36
- PEDW (Patient Episode Database for Wales) ... 37
- SACT (Systematic Anti Cancer Therapy) ... 38
- SMDS (Substance Misuse DataSet) ... 39
- SWAC (School Workforce Annual Census) ... 40
- WASD (Welsh Ambulance Service Dataset) ... 44
- WLGP (Wales Longitudinal General Practice) ... 44

Supplementary Table 3– meta-data of existing ethnicity RRDA tables ... 50
Supplementary Table 4– characteristics of the cohort based on ONS categorisation. ... 52
Supplementary Table 5 – characteristics of the cohort based on NER categorisation. ... 52

Supplementary Figure 1-longitudinal coverage of contributing data sources into ethnicity spine

Ethnicity records have been extracted from Electronic Health Records (EHRs). While multiple data sources have contributed to achieving an accurate ethnic group for each individual, we depict contribution level of each data source over time in the figure A (contribution level) and B (data source range).

Figure 1-A

A- Contribution level of each data source: No prioritisation was applied; records from every data source have **equal value**; we sequentially remove high-contributing data sources to drill down on the data sources contributing to the ethnic group over time.

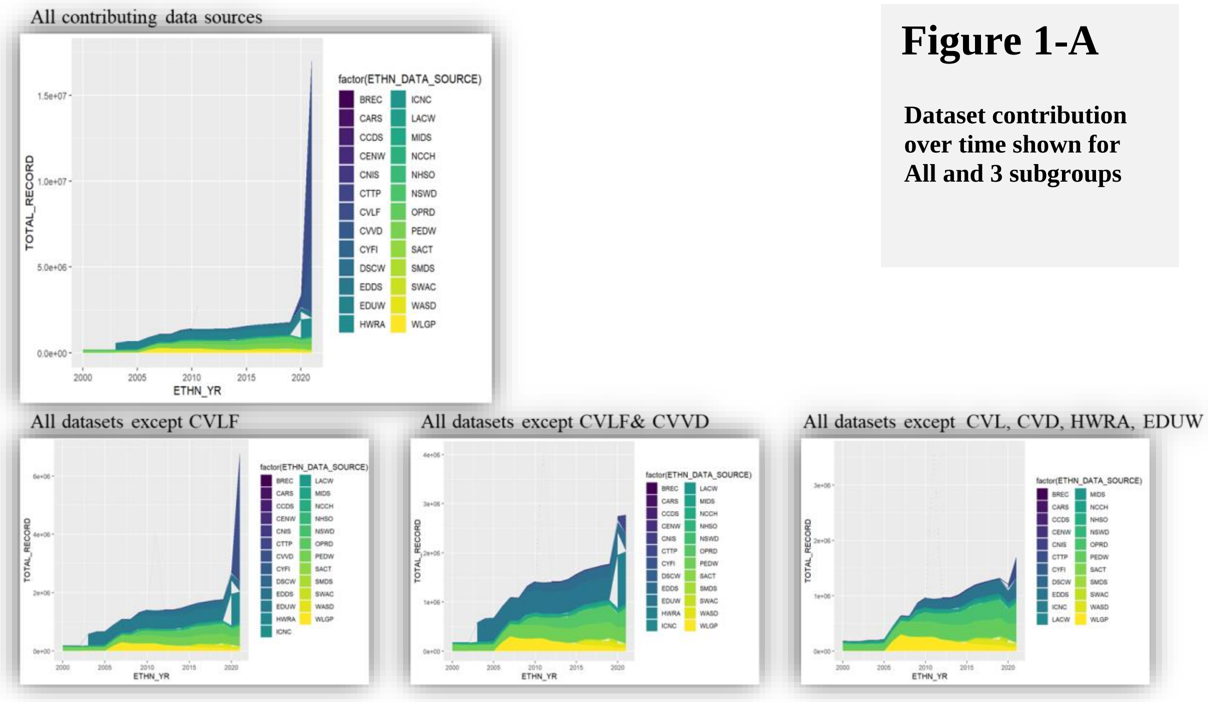

Figure 1-B

B- Dataset range over time: x axis for all datasets are set to *2000 and 2020 so lack of line means there is not data and existence of the line means start point of the dataset.*

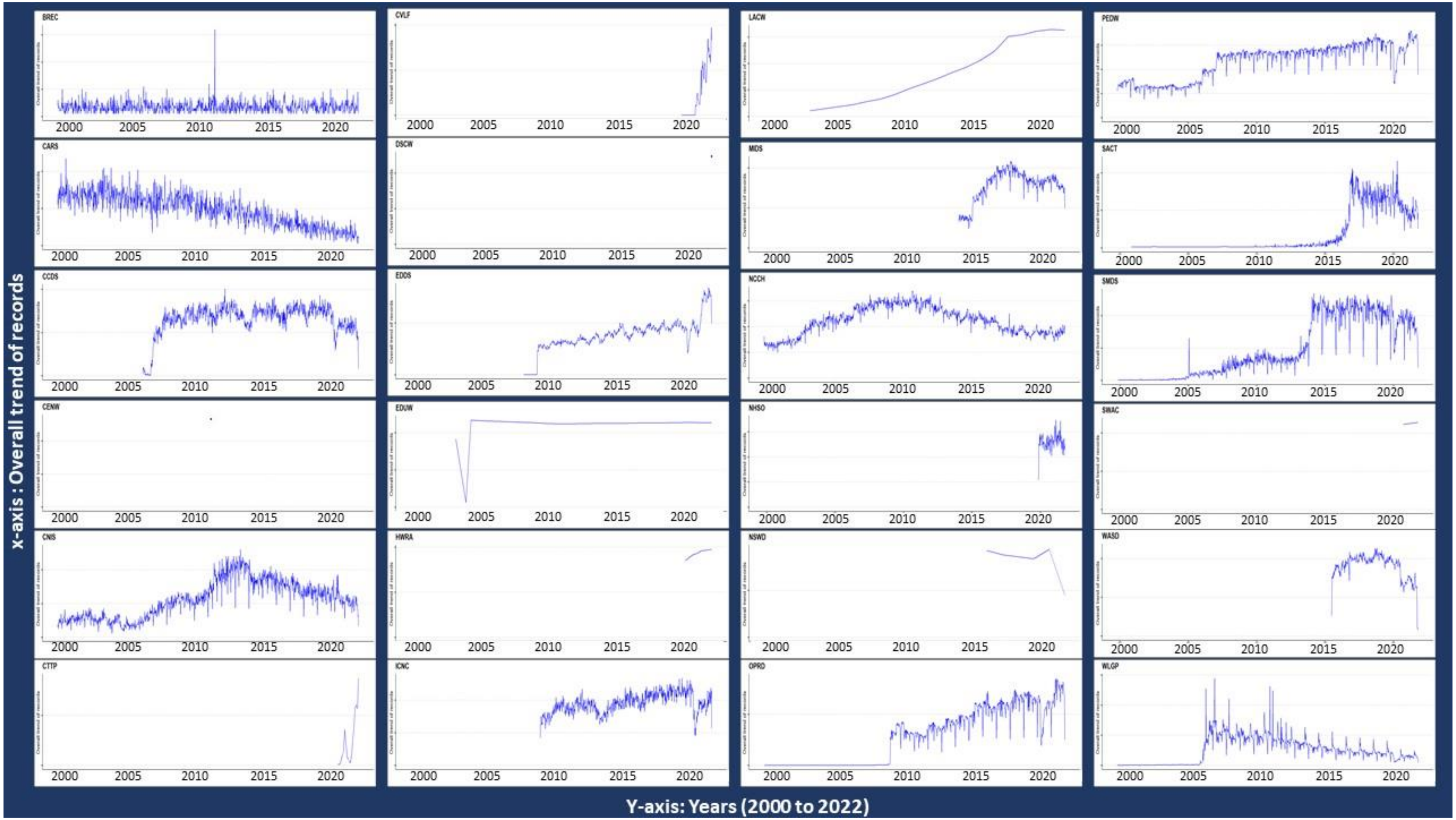

Supplementary Figure 2– *distribution of ethnic groups across sex & age groups for ONS categorisation for four different retrieval method approaches.*

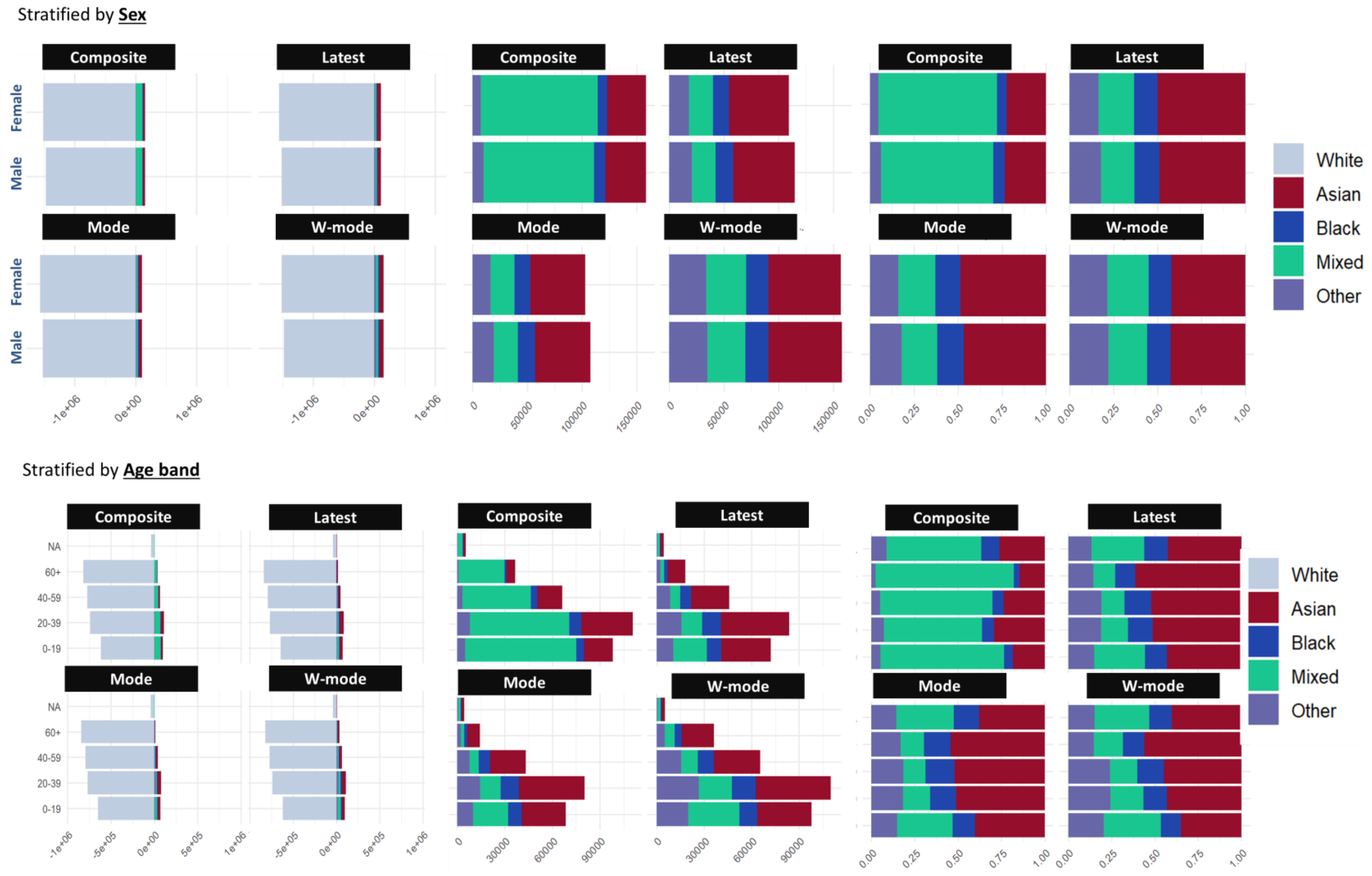

Supplementary Figure 3 – *distribution of ethnic groups across sex & age groups for NER categorisation for four different retrieval method approaches.*

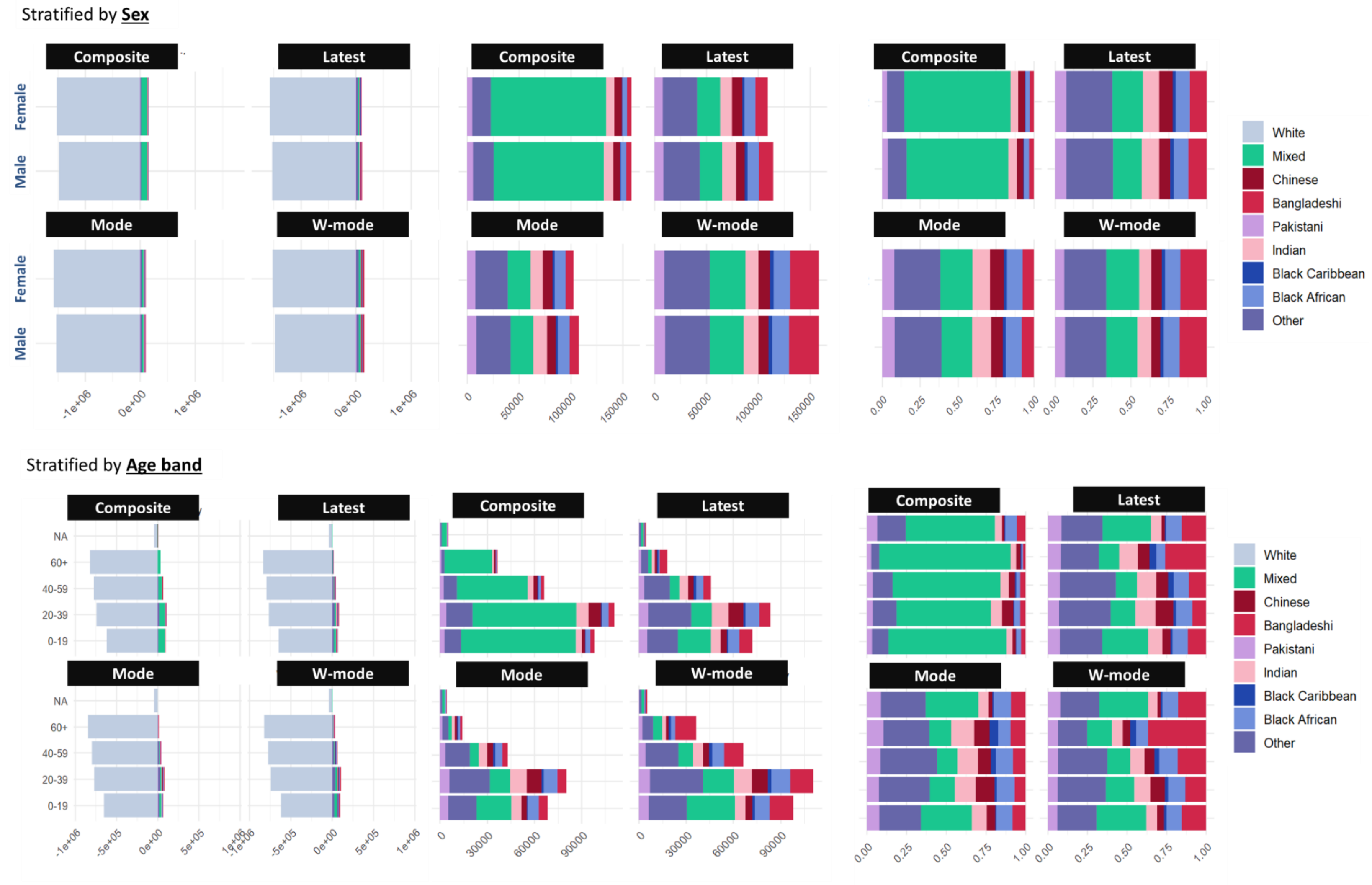

Supplementary Figure 4 – changes in population over time since 2011 (x-axis shows days since 2011-01-01)

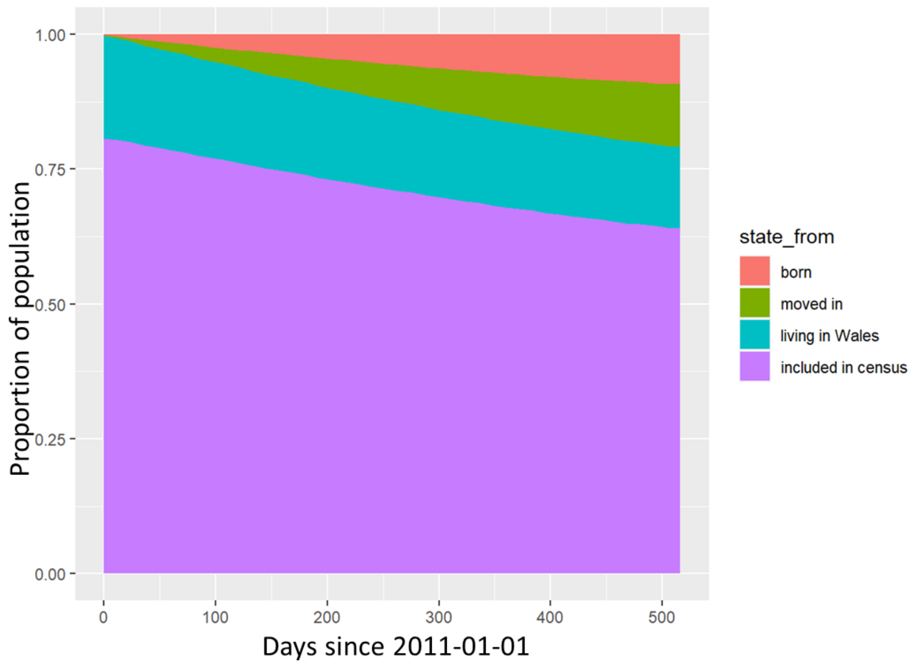

Supplementary Figure 5 – kappa index between all longitudinal records and Census data

(Please note: The kappa of a dataset with itself has demonstrated to a less than 1.00 where individuals had contradicting records over time in a single dataset with itself). This figure demonstrates the level of agreement in ethnicity records across two datasets. The agreement level are colour codes with lighter shades being 100% match of records across two datasets contributing to that cell and dark blue shade being 0% or minimal agreement of ethnicity records across two datasets.

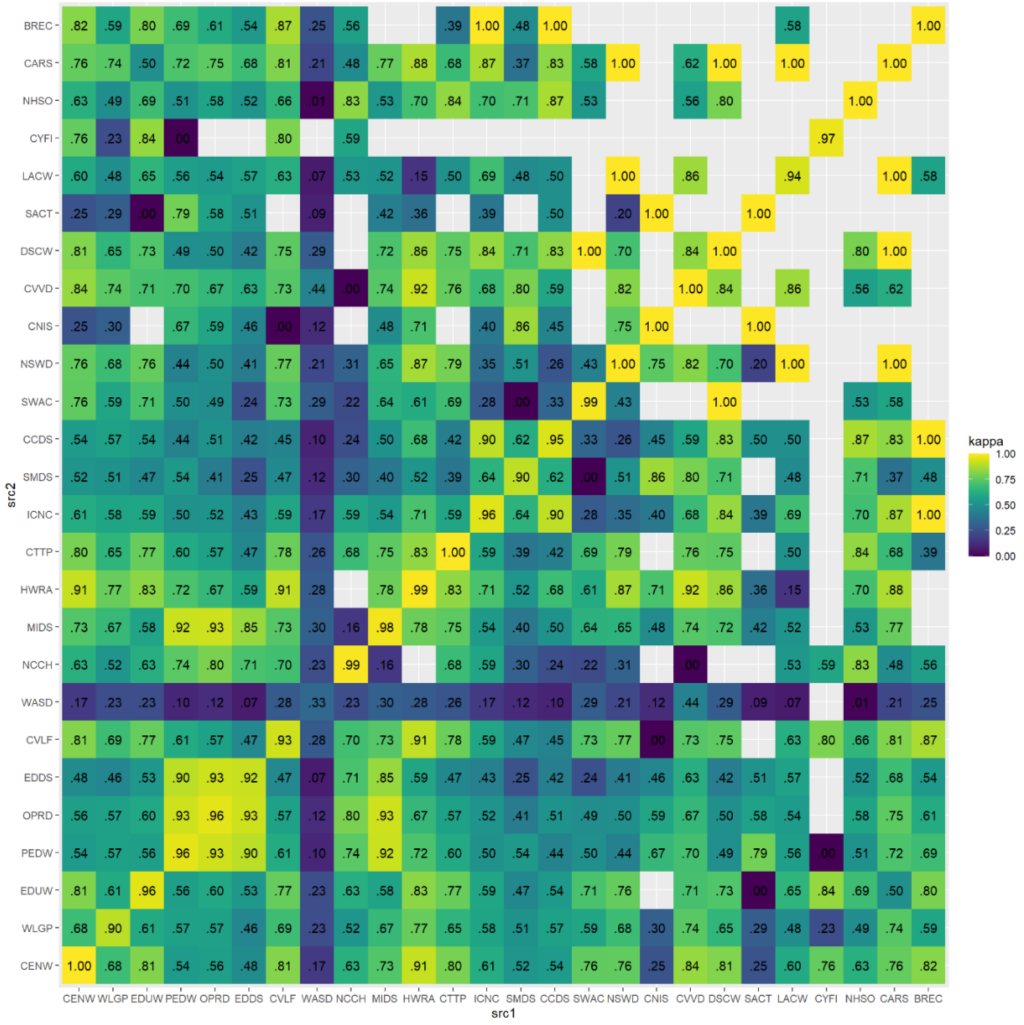

Cohen's Kappa	Interpretation
0	No agreement
0.10-0.20	Slight agreement
0.21-0.40	Fair agreement
0.41-0.60	Moderate agreement
0.61-0.80	Substantial agreement
0.81-0.99	Near perfect agreement
1	100% match

Supplementary Table 1 – name and coverage of data sources used in the creation of ethnicity spine for Wales

Data flow	Data Source	Full name
Daily	CTTP	Contact Tracking Trace and Protect
Daily	CVLF	COVID-19 Lateral Flow test
Daily	CVVD	COVID Vaccine Data
Daily	NHSO	NHS 111 Call data
Daily	SACT	Systemic Anti-Cancer Therapy
Daily	WASD	Welsh Ambulance Service Dataset
Weekly	CNIS	Cancer Network Information System Cymru (CaNISC)
Weekly	PEDW	Patient Episode Database for Wales
Quarterly	ICNC	ICNARC – Intensive Care National Audit & Research Centre
Quarterly	NCCH	National Community Child Health database
One-off	CENW	Office for National Statistics 2011 Census
Monthly	CCDS	Critical Care Data Set
Monthly	EDDS	Emergency Department Data Set
Monthly	MIDS	Maternity and childbirth Indicator DataSet
Monthly	HWRA	Healthcare Workers Risk Assessment
Monthly	OPRD	OutPatient Referral Dataset
Monthly	SMDS	Substance Misuse Dataset
Monthly	WLGP	Welsh Longitudinal General Practice
Yearly	BREC	Brecon dataset
Yearly	CARS	Congenital Anomaly Register and Information Services for Wales
Yearly	CYFI	Cystic Fibrosis Registry
Yearly	DSCW	Domiciliary Social Care Workers
Yearly	EDUW	Education data on schools and pupils
Yearly	LACW	Looked After Children Wales
Yearly	SWAC	School Workforce Annual Census
One-off	NSWD	National Survey for Wales Dataset

Supplementary Table 2 - Ethnic group record extraction details

The following tables provide details on data sources and categorisation and harmonisation rules used to extract the records.

https://portal.caliberresearch.org/phenotypes/ethnic-status

BREC (Brecon data)

ONS category	Data source code	Data source description
**1 White**		'any other white background'<br>'british'<br>'irish'
**2 Mixed**		'any other mixed background'<br>'white and asian'<br>'white and black african'<br>'white and black caribbean'
**3 Asian**		'any other asian background'<br>'asian'<br>'bangladeshi'<br>'chinese'<br>'indian'<br>'pakistani'
**4 Black**		'african'<br>'any other black background'<br>'black african'<br>'caribbean'
**5 Other**		'any other ethnic group'

NER category	Data source code	Data source description
**1 White**		'any other white background'<br>'british'<br>'irish'
**2 Mixed**		'any other mixed background'<br>'white and asian'<br>'white and black african'<br>'white and black caribbean'
**3 Indian**		'indian'
**4 Pakistani**		'pakistani'
**5 Bangladeshi**		'bangladeshi'
**6 Chinese**		'chinese'
**7 Black Caribbean**		'caribbean'
**8 Black African**		'african'<br>'black african'
**9 Other**		'any other asian background'<br>'any other black background'<br>'any other ethnic group'<br>'asian'

CARS (Congenital Anomaly Register and Information Services for Wales)

ONS category	Data source code	Data source description
**1 White**	1<br>2<br>3	White British<br>White Irish<br>White any other
**2 Mixed**	4<br>5<br>6<br>7	Mixed W& B Car<br>Mixed W & B Afr<br>Mixed W & Asian<br>Mixed any other
**3 Asian**	8<br>10<br>11<br>12<br>18	Indian<br>Pakistani<br>Bangladeshi<br>Asian, other<br>Other, Chinese
**4 Black**	13<br>14<br>16	Black Caribbean<br>Black African<br>Black, other
**5 Other**	19	Other, any other

NER category	Data source code	Data source description
**1 White**	1<br>2<br>3	White British<br>White Irish<br>White any other
**2 Mixed**	4<br>5<br>6<br>7	Mixed W& B Car<br>Mixed W & B Afr<br>Mixed W & Asian<br>Mixed any other
**3 Indian**	8	Indian
**4 Pakistani**	10	Pakistani
**5 Bangladeshi**	11	Bangladeshi
**6 Chinese**	18	Other, Chinese
**7 Black Caribbean**	13	Black Caribbean
**8 Black African**	14	Black African
**9 Other**	16<br>19	Asian, other<br>Black, other

CCDS (Critical Care DataSet)

ONS category	Data source code	Data source description
**1 White**	A<br><br>B	Any White Background, including Welsh, English, Scottish, Northern Irish, Irish, British<br>Gypsy or Irish Traveller
**2 Mixed**	D<br>E<br>F<br>G	White and Black Caribbean<br>White and Black African<br>White and Asian<br>Any other mixed background / multiple ethnic background
**3 Asian**	H<br>J<br>K<br>L<br>R	Indian<br>Pakistani<br>Bangladeshi<br>Any other Asian Background<br>Chinese
**4 Black**	M<br>N<br>P	Caribbean<br>African<br>Any other Black background
**5 Other**	S<br>T	Any other ethnic group<br>Arab

NER category	Data source code	Data source description
**1 White**	A<br><br>B	Any White Background, including Welsh, English, Scottish, Northern Irish, Irish, British<br>Gypsy or Irish Traveller
**2 Mixed**	D<br>E<br>F<br>G	White and Black Caribbean<br>White and Black African<br>White and Asian<br>Any other mixed background / multiple ethnic background
**3 Indian**	H	Indian
**4 Pakistani**	J	Pakistani
**5 Bangladeshi**	K	Bangladeshi
**6 Chinese**	R	Chinese
**7 Black Caribbean**	M	Caribbean
**8 Black African**	N	African
**9 Other**	L<br>P<br>S<br>T	Any other Asian Background<br>Any other Black background<br>Any other ethnic group<br>Arab

CENW (ONS Census 2011)

ONS category	Data source code	Data source description
**1 White**	01	English / Welsh / Scottish / Northern Irish / British
	02	Irish
	03	Gypsy or Irish Traveller
	04	Other White
**2 Mixed**	05	White and Black Caribbean
	06	White and Black African
	07	White and Asian
	08	Other Mixed
**3 Asian**	09	Indian
	10	Pakistani
	11	Bangladeshi
	12	Chinese
	13	Other Asian
**4 Black**	14	African
	15	Caribbean
	16	Other Black
**5 Other**	17	Arab
	18	Other Ethnic Group

NER category	Data source code	Data source description
**1 White**	01	English / Welsh / Scottish / Northern Irish / British
	02	Irish
	03	Gypsy or Irish Traveller
	04	Other White
**2 Mixed**	05	White and Black Caribbean
	06	White and Black African
	07	White and Asian
	08	Other Mixed
**3 Indian**	09	Indian
**4 Pakistani**	10	Pakistani
**5 Bangladeshi**	11	Bangladeshi
**6 Chinese**	12	Chinese
**7 Black Caribbean**	14	African
**8 Black African**	15	Caribbean
**9 Other**	13	Other Asian
	16	Other Black
	17	Arab
	18	Other Ethnic Group

CNIS (CANISC – Cancer Network Information System Cymru)

ONS category	Data source code	Data source description
**1 White**	A<br><br>B	Any White Background, including Welsh, English, Scottish, Northern Irish, Irish, British<br>Gypsy or Irish Traveller
**2 Mixed**	D<br>E<br>F<br>G	White and Black Caribbean<br>White and Black African<br>White and Asian<br>Any other mixed background / multiple ethnic background
**3 Asian**	H<br>J<br>K<br>L<br>R	Indian<br>Pakistani<br>Bangladeshi<br>Any other Asian Background<br>Chinese
**4 Black**	M<br>N<br>P	Caribbean<br>African<br>Any other Black background
**5 Other**	S<br>T	Any other ethnic group<br>Arab

NER category	Data source code	Data source description
**1 White**	A<br><br>B	Any White Background, including Welsh, English, Scottish, Northern Irish, Irish, British<br>Gypsy or Irish Traveller
**2 Mixed**	D<br>E<br>F<br>G	White and Black Caribbean<br>White and Black African<br>White and Asian<br>Any other mixed background / multiple ethnic background
**3 Indian**	H	Indian
**4 Pakistani**	J	Pakistani
**5 Bangladeshi**	K	Bangladeshi
**6 Chinese**	R	Chinese
**7 Black Caribbean**	M	Caribbean
**8 Black African**	N	African
**9 Other**	L<br>P<br>S<br>T	Any other Asian Background<br>Any other Black background<br>Any other ethnic group<br>Arab

121 CTTP (COVID-19 Test Trace and Protect)

ONS category	Data source code	Data source description
**1 White**		'Albanian'<br>'Any other White background'<br>'Any White background'<br>'Bulgarian'<br>'Croatian'<br>'Czech'<br>'English, Welsh, Scottish, Northern Irish or British'<br>'EU Roma'<br>'French'<br>'German'<br>'Greek/Greek Cypriot'<br>'Gypsy or Irish Traveller'<br>'Hungarian'<br>'Irish'<br>'Italian'<br>'Latvian'<br>'Lithuanian'<br>'Maltese'<br>'Other White'<br>'Polish'<br>'Portuguese'<br>'Roma'<br>'Romanian'<br>'Russian'<br>'Scandinavian'<br>'Serbian'<br>'Slovakian'<br>'Slovenian'<br>'Spanish'<br>'Turkish/Turkish Cypriot'<br>'Ukrainian'<br>'White ? British'<br>'White European Other’
**2 Mixed**		'Any Other Mixed Background'<br>'Any other mixed background'<br>'Any other Mixed/Multiple background'<br>'Black And Any Other Ethnic Group'<br>'Chinese And Any Other Ethnic Group'<br>'Other Mixed Background'<br>'White And Any Other Ethnic Group'<br>'White and Asian'<br>'White and Black African'<br>'White and Black Caribbean'
**3 Asian**		'Any other Asian background'<br>'Asian And Any Other Ethnic Group'<br>'Asian And Chinese'<br>'Bangladeshi'<br>'Chinese'<br>'Hong Kong Chinese'<br>'Indian'<br>'Japanese'<br>'Korean'<br>'Malaysian Chinese'<br>'Nepali'<br>'Other Asian'<br>'Other Pakistani'<br>'Pakistani'<br>'Sinhalese'<br>'Sri Lankan Tamil'<br>'Thai'
**4 Black**		'African'<br>'Any other Black background'<br>'Any other Black or African or Caribbean background'<br>'Black European'<br>'Black North American'<br>'Caribbean'<br>'Ghanaian'<br>'Nigerian'<br>'Other Black African'<br>'Somali'<br>'Sudanese'

**5 Other**		'Any other ethnic background'<br>'Any other ethnic group'<br>'Arab'<br>'Egyptian'<br>'Filipino'<br>'Irani'<br>'Iraqi'<br>'Kurdish'<br>'Latin/South/Central American'<br>'Lebanese'<br>'Libyan'<br>'Malay'<br>'Moroccan'<br>'Other Ethnic Group'<br>'Polynesian'<br>'Saudi Arabian'<br>'Syrian'<br>'Vietnamese'<br>'Yemeni'

122

NER category	Data source code	Data source description
**1 White**		'Albanian'<br>'Any other White background'<br>'Any White background'<br>'Bulgarian'<br>'Croatian'<br>'Czech'<br>'English, Welsh, Scottish, Northern Irish or British'<br>'EU Roma'<br>'French'<br>'German'<br>'Greek/Greek Cypriot'<br>'Gypsy or Irish Traveller'<br>'Hungarian'<br>'Irish'<br>'Italian'<br>'Latvian'<br>'Lithuanian'<br>'Maltese'<br>'Other White'<br>'Polish'<br>'Portuguese'<br>'Roma'<br>'Romanian'<br>'Russian'<br>'Scandinavian'<br>'Serbian'<br>'Slovakian'<br>'Slovenian'<br>'Spanish'<br>'Turkish/Turkish Cypriot'<br>'Ukrainian'<br>'White ? British'<br>'White European Other’
**2 Mixed**		'Any Other Mixed Background'<br>'Any other mixed background'<br>'Any other Mixed/Multiple background'<br>'Black And Any Other Ethnic Group'<br>'Chinese And Any Other Ethnic Group'<br>'Other Mixed Background'<br>'White And Any Other Ethnic Group'<br>'White and Asian'<br>'White and Black African'<br>'White and Black Caribbean'
**3 Indian**		‘Indian’
**4 Pakistani**		'Other Pakistani'<br>'Pakistani'
**5 Bangladeshi**		‘Bangladeshi’
**6 Chinese**		'Asian And Chinese'<br>'Chinese'<br>'Hong Kong Chinese'<br>'Malaysian Chinese'

**7 Black Caribbean**		‘Caribbean’
**8 Black African**		'African'<br>'Ghanaian'<br>'Nigerian'<br>'Other Black African'<br>'Somali'<br>'Sudanese'
**9 Other**		'Any other Asian background'<br>'Any other Black background'<br>'Any other Black or African or Caribbean background'<br>'Any other ethnic background'<br>'Any other ethnic group'<br>'Arab'<br>'Asian And Any Other Ethnic Group'<br>'Asian And Chinese'<br>'Black European'<br>'Black North American'<br>'Egyptian'<br>'Filipino'<br>'Irani'<br>'Iraqi'<br>'Japanese'<br>'Korean'<br>'Kurdish'<br>'Latin/South/Central American'<br>'Lebanese'<br>'Libyan'<br>'Malay'<br>'Moroccan'<br>'Nepali'<br>'Other Asian'<br>'Other Ethnic Group'<br>'Polynesian'<br>'Saudi Arabian'<br>'Sinhalese'<br>'Sri Lankan Tamil'<br>'Syrian'<br>'Thai'<br>'Vietnamese'<br>'Yemeni'

CVLF (COVID-19 Lateral Flow)

ONS category	Data source code	Data source description
**1 White**		'Another White background'<br>'British~ English~ Northern Irish~ Scottish~ or Welsh'<br>'Irish Traveller or Gypsy'<br>'Irish'<br>'White'
**2 Mixed**		'Another Mixed background'<br>'Asian and White'<br>'Black African and White'<br>'Black Caribbean and White'<br>'Mixed or multiple ethnic groups'
**3 Asian**		'Another Asian background'<br>'Asian or Asian British'<br>'Bangladeshi'<br>'Chinese'<br>'Indian'<br>'Pakistani'
**4 Black**		'African'<br>'Another Black background'<br>'Black~ African~ Black British or Caribbean'<br>'Caribbean'
**5 Other**		'Another ethnic background'<br>'Another ethnic group'<br>'Arab'

NER category	Data source code	Data source description
**1 White**		'Another White background'<br>'British~ English~ Northern Irish~ Scottish~ or Welsh'<br>'Irish Traveller or Gypsy'<br>'Irish'<br>'White'
**2 Mixed**		'Another Mixed background'<br>'Asian and White'<br>'Black African and White'<br>'Black Caribbean and White'<br>'Mixed or multiple ethnic groups'
**3 Indian**		'Indian'
**4 Pakistani**		'Pakistani'
**5 Bangladeshi**		'Bangladeshi'
**6 Chinese**		'Chinese'
**7 Black Caribbean**		'Caribbean'
**8 Black African**		'African'
**9 Other**		'Another Asian background'<br>'Another Black background'<br>'Another ethnic background'<br>'Another ethnic group'<br>'Arab'<br>'Asian or Asian British'<br>'Black~ African~ Black British or Caribbean'

CVVD (COVID-19 Vaccine Data)

ONS category	Data source code	Data source description
**1 White**	A<br><br>B	Any White Background, including Welsh, English, Scottish, Northern Irish, Irish, British<br>Gypsy or Irish Traveller
**2 Mixed**	D<br>E<br>F<br>G	White and Black Caribbean<br>White and Black African<br>White and Asian<br>Any other mixed background / multiple ethnic background
**3 Asian**	H<br>J<br>K<br>L<br>R	Indian<br>Pakistani<br>Bangladeshi<br>Any other Asian Background<br>Chinese
**4 Black**	M<br>N<br>P	Caribbean<br>African<br>Any other Black background
**5 Other**	S<br>T	Any other ethnic group<br>Arab

NER category	Data source code	Data source description
**1 White**	A<br><br>B	Any White Background, including Welsh, English, Scottish, Northern Irish, Irish, British<br>Gypsy or Irish Traveller
**2 Mixed**	D<br>E<br>F<br>G	White and Black Caribbean<br>White and Black African<br>White and Asian<br>Any other mixed background / multiple ethnic background
**3 Indian**	H	Indian
**4 Pakistani**	J	Pakistani
**5 Bangladeshi**	K	Bangladeshi
**6 Chinese**	R	Chinese
**7 Black Caribbean**	M	Caribbean
**8 Black African**	N	African
**9 Other**	L<br>P<br>S<br>T	Any other Asian Background<br>Any other Black background<br>Any other ethnic group<br>Arab

CYFI (Cystic Fibrosis Register)

ONS category	Data source code	Data source description
**1 White**		'WBritish', 'WIrish', 'WOther'
**2 Mixed**		‘MWBC', 'MWAs', 'MWBA', 'Mother’
**3 Asian**		‘ABangladeshi', 'AIndian', 'AOther', 'APakistani', 'OChinese'
**4 Black**		'BAfrican', 'BCaribbean', 'BOther'
**5 Other**		'OOther'

NER category	Data source code	Data source description
**1 White**		'WBritish', 'WIrish', 'WOther'
**2 Mixed**		‘MWBC', 'MWAs', 'MWBA', 'Mother’
**3 Indian**		'AIndian'
**4 Pakistani**		'APakistani'
**5 Bangladeshi**		‘ABangladeshi'
**6 Chinese**		'OChinese'
**7 Black Caribbean**		'BCaribbean'
**8 Black African**		'BAfrican',
**9 Other**		'AOther', 'BOther', 'OOther'

137 DSCW (Domiciliary Social Care Wales)

ONS category	Data source code	Data source description
**1 White**		'Any other White background, please state'<br>'European'<br>'Irish traveller'<br>'Welsh'<br>'White'<br>'White British'<br>'White English'<br>'White Irish'<br>'White Welsh'<br>'White or White British'<br>'White welsh'<br>'white welsh'
**2 Mixed**		'Any other mixed background'<br>'Mixed White and Asian'<br>'Mixed White and Black African'<br>'Mixed White and Black Caribbean'<br>'Mixed ethnic group'
**3 Asian**		'Any other Asian background, please state'<br>'Any other Chinese background'<br>'Asian or Asian British Bangladeshi'<br>'Asian or Asian British Indian'<br>'Asian or Asian British Pakistani'<br>'Bangladeshi'<br>'Chinese'<br>'Chinese British'<br>'Chinese or Chinese British Chinese'<br>'Indian'<br>'Pakistani'
**4 Black**		'Any other Black background, please state'<br>'Black African'<br>'Black Caribbean'<br>'Black or Black British African'<br>'Black or Black British Caribbean'
**5 Other**		'Any Other'<br>'Any other ethnic background, please state'<br>'Other Ethnicity'

138

NER category	Data source code	Data source description
**1 White**		'Any other White background, please state'<br>'European'<br>'Irish traveller'<br>'Welsh'<br>'White'<br>'White British'<br>'White English'<br>'White Irish'<br>'White Welsh'<br>'White or White British'<br>'White welsh'<br>'white welsh'
**2 Mixed**		'Any other mixed background'<br>'Mixed White and Asian'<br>'Mixed White and Black African'<br>'Mixed White and Black Caribbean'<br>'Mixed ethnic group'
**3 Indian**		'Asian or Asian British Indian'<br>'Indian'
**4 Pakistani**		'Asian or Asian British Pakistani'<br>'Pakistani'
**5 Bangladeshi**		'Asian or Asian British Bangladeshi'<br>'Bangladeshi'
**6 Chinese**		'Any other Chinese background'<br>'Chinese'<br>'Chinese British'<br>'Chinese or Chinese British Chinese'
**7 Black Caribbean**		'Black Caribbean'<br>'Black or Black British Caribbean'
**8 Black African**		Black African'<br>'Black or Black British African'

**9 Other**		'Any Other'<br>'Any other Asian background, please state'<br>'Any other Black background, please state'<br>'Any other ethnic background, please state'<br>'Other Ethnicity'

EDDS (Emergency Department DataSet)

ONS category	Data source code	Data source description
**1 White**	0<br>A<br><br>B	White<br>Any White Background, including Welsh, English, Scottish, Northern Irish, Irish, British<br>Gypsy or Irish Traveller
**2 Mixed**	D<br>E<br>F<br>G	White and Black Caribbean<br>White and Black African<br>White and Asian<br>Any other mixed background / multiple ethnic background
**3 Asian**	4<br>5<br>6<br>7<br>H<br>J<br>K<br>L<br>R	Indian<br>Pakistani<br>Bangladeshi<br>Chinese<br>Indian<br>Pakistani<br>Bangladeshi<br>Any other Asian Background<br>Chinese
**4 Black**	1<br>2<br>3<br>M<br>N<br>P	Black - Caribbean<br>Black - African<br>Black - Other<br>Caribbean<br>African<br>Any other Black background
**5 Other**	8<br>S<br>T	Any other ethnic group<br>Any other ethnic group<br>Arab

NER category	Data source code	Data source description
**1 White**	0<br>A<br><br>B	White<br>Any White Background, including Welsh, English, Scottish, Northern Irish, Irish, British<br>Gypsy or Irish Traveller
**2 Mixed**	D<br>E<br>F<br>G	White and Black Caribbean<br>White and Black African<br>White and Asian<br>Any other mixed background / multiple ethnic background
**3 Indian**	4<br>H	Indian<br>Indian
**4 Pakistani**	5<br>J	Pakistani<br>Pakistani
**5 Bangladeshi**	6<br>K	Bangladeshi<br>Bangladeshi
**6 Chinese**	7<br>R	Chinese<br>Chinese
**7 Black Caribbean**	1<br>M	Black - Caribbean<br>Caribbean
**8 Black African**	2<br>N	Black - African<br>African
**9 Other**	3<br>8<br>L<br>P<br>S<br>T	Black - Other<br>Any other ethnic group<br>Any other Asian Background<br>Any other Black background<br>Any other ethnic group<br>Arab

145 EDUW (Education data)

ONS category	Data source code	Data source description
**1 White**	WALB	Albanian
	WBGR	British Gypsy / Gypsy Roma
	WBOS	Bosnian-Herzegovinian
	WBRI	White – British
	WBUL	Bulgarian
	WCRO	Croatian
	WCZE	Czech
	WEEU	White Eastern European
	WEUR	White European Other
	WFRE	French
	WGER	German
	WGRE	Greek / Greek Cypriot
	WGRO	Gypsy / Gypsy Roma from Other Countries
	WHUN	Hungarian
	WITA	Italian
	WITH	Traveller of Irish Heritage
	WIRT	Traveller
	WKOS	Kosovan
	WLAT	Latvian
	WLIT	Lithuanian
	WMAL	Maltese
	WMON	Montenegran
	WNAG	New Traveller
	WOBG	British Gypsy
	WOCC	Occupational Traveller
	WOER	EU Roma
	WOGR	Other Gypsy / Gypsy Roma
	WOOG	Gypsy from Other Countries
	WOOR	Roma from Other Countries
	WOTG	Other Gypsy
	WOTH	Any other White Background
	WOTR	Other Roma
	WOTT	Other Traveller
	WOTW	Other White
	WPOL	Polish
	WPOR	Portuguese
	WRMA	Romanian
	WROM	Gypsy / Gypsy Roma
	WRUS	Russian
	WSCA	Scandinavian
	WSER	Serbian
	WSPA	Spanish
	WSVK	Slovakian
	WSVN	Slovenian
	WTUR	Turkish / Turkish Cypriot
	WUKR	Ukrainian
	WWEU	White Western European
**2 Mixed**	MABL	Asian and Black
	MACH	Asian and Chinese
	MAOE	Asian and Any Other Ethnic Group
	MBCH	Black and Chinese
	MBOE	Black and Other Ethnic Group
	MCOE	Chinese and Any Other Ethnic Group
	MOTH	Any Other Mixed Background
	MOTM	Other Mixed Background
	MWAS	White and Asian
	MWBA	White and Black African
	MWBC	White and Black Caribbean
	MWCH	White and Chinese
	MWOE	White and Any Other Ethnic Group
**3 Asian**	AAFR	African Asian
	ABAN	Bangladeshi
	AIND	Indian
	AKAS	Kashmiri
	AMPK	Mirpuri Pakistani
	ANEP	Nepali
	AOTA	Other Asian
	AOTH	Any Other Asian Background
	APOK	Other Pakistani
	APKN	Pakistani
	ASNL	Sinhalese
	ASLT	Sri Lankan Tamil

ONS category	Data source code	Data source description
	CHKC	Hong Kong Chinese
	CHNE	Chinese or Chinese British
	CMAL	Malaysian Chinese
	CSNG	Singaporean Chinese
	CTWN	Taiwanese
	OFIL	Filipino
	OJPN	Japanese
	OKOR	Korean
	OMAL	Malay
	OOCH	Other Chinese
**4 Black**	BAFR	African
	BAOF	Other Black African
	BCRB	Caribbean
	BERI	Eritrean
	BEUR	Black European
	BGHA	Ghanaian
	BNAM	Black North American
	BOTB	Other Black
	BOTH	Any other Black background
	BNGN	Nigerian
	BSLN	Sierra Leonian
	BSOM	Somali
	BSUD	Sudanese
**5 Other**	OAFG	Afghanistani
	OARA	Arab
	OEGY	Egyptian
	OIRN	Iranian
	OIRQ	Iraqi
	OKRD	Kurdish
	OLAM	Latin / South / Central American
	OLEB	Lebanese
	OLIB	Libyan
	OMRC	Moroccan
	OOEG	Other ethnic group
	OOTH	Any other ethnic background
	OPOL	Polynesian
	OSYR	Syrian
	OTHA	Thai
	OSAU	Saudi Arabian
	OVIE	Vietnamese
	OYEM	Yemeni

146

NER category	Data source code	Data source description
**1 White**	WALB	Albanian
	WBGR	British Gypsy / Gypsy Roma
	WBOS	Bosnian-Herzegovinian
	WBRI	White – British
	WBUL	Bulgarian
	WCRO	Croatian
	WCZE	Czech
	WEEU	White Eastern European
	WEUR	White European Other
	WFRE	French
	WGER	German
	WGRE	Greek / Greek Cypriot
	WGRO	Gypsy / Gypsy Roma from Other Countries
	WHUN	Hungarian
	WITA	Italian
	WITH	Traveller of Irish Heritage
	WIRT	Traveller
	WKOS	Kosovan
	WLAT	Latvian
	WLIT	Lithuanian
	WMAL	Maltese
	WMON	Montenegran
	WNAG	New Traveller
	WOBG	British Gypsy
	WOCC	Occupational Traveller
	WOER	EU Roma
	WOGR	Other Gypsy / Gypsy Roma
	WOOG	Gypsy from Other Countries
	WOOR	Roma from Other Countries
	WOTG	Other Gypsy

NER category	Data source code	Data source description
	WOTH<br>WOTR<br>WOTT<br>WOTW<br>WPOL<br>WPOR<br>WRMA<br>WROM<br>WRUS<br>WSCA<br>WSER<br>WSPA<br>WSVK<br>WSVN<br>WTUR<br>WUKR<br>WWEU	Any other White Background<br>Other Roma<br>Other Traveller<br>Other White<br>Polish<br>Portuguese<br>Romanian<br>Gypsy / Gypsy Roma<br>Russian<br>Scandinavian<br>Serbian<br>Spanish<br>Slovakian<br>Slovenian<br>Turkish / Turkish Cypriot<br>Ukrainian<br>White Western European
**2 Mixed**	MABL<br>MACH<br>MAOE<br>MBCH<br>MBOE<br>MCOE<br>MOTH<br>MOTM<br>MWAS<br>MWBA<br>MWBC<br>MWCH<br>MWOE	Asian and Black<br>Asian and Chinese<br>Asian and Any Other Ethnic Group<br>Black and Chinese<br>Black and Other Ethnic Group<br>Chinese and Any Other Ethnic Group<br>Any Other Mixed Background<br>Other Mixed Background<br>White and Asian<br>White and Black African<br>White and Black Caribbean<br>White and Chinese<br>White and Any Other Ethnic Group
**3 Indian**	AIND	Indian
**4 Pakistani**	AMPK<br>AOPK<br>APKN	Mirpuri Pakistani<br>Other Pakistani<br>Pakistani
**5 Bangladeshi**	ABAN	Bangladeshi
**6 Chinese**	CHKC<br>CHNE<br>CMAL<br>CSNG<br>CTWN<br>OOCH	Hong Kong Chinese<br>Chinese or Chinese British<br>Malaysian Chinese<br>Singaporean Chinese<br>Taiwanese<br>Other Chinese
**7 Black Caribbean**	BCRB	Caribbean
**8 Black African**	BAFR<br>BAOF<br>BERI<br>BGHA<br>BNGN<br>BSLN<br>BSOM<br>BSUD	African<br>Other Black African<br>Eritrean<br>Ghanaian<br>Nigerian<br>Sierra Leonian<br>Somali<br>Sudanese
**9 Other**	AAFR<br>AKAS<br>ANEP<br>AOTA<br>AOTH<br>ASNL<br>ASLT<br>BEUR<br>BNAM<br>BOTB<br>BOTH<br>OAFG<br>OARA<br>OEGY<br>OFIL<br>OIRN<br>OIRQ<br>OJPN<br>OKOR<br>OKRD<br>OLAM<br>OLEB<br>OLIB<br>OMAL	African Asian<br>Kashmiri<br>Nepali<br>Other Asian<br>Any Other Asian Background<br>Sinhalese<br>Sri Lankan Tamil<br>Black European<br>Black North American<br>Other Black<br>Any other Black background<br>Afghanistani<br>Arab<br>Egyptian<br>Filipino<br>Iranian<br>Iraqi<br>Japanese<br>Korean<br>Kurdish<br>Latin / South / Central American<br>Lebanese<br>Libyan<br>Malay

NER category	Data source code	Data source description
	OOEG	Other ethnic group
	OOTH	Any other ethnic background
	OPOL	Polynesian
	OSAU	Saudi Arabian
	OSYR	Syrian
	OTHA	Thai
	OVIE	Vietnamese
	OYEM	Yemeni

HWRA (Health Worker Risk Assessment)

ONS category	Data source code	Data source description
**1 White**		'A White - British'<br>'B White - Irish'<br>'C White - Any other White background'<br>'C2 White Northern Irish'<br>'C3 White Unspecified'<br>'CA White English'<br>'CB White Scottish'<br>'CC White Welsh'<br>'CD White Cornish'<br>'CE White Cypriot (non specific)'<br>'CF White Greek'<br>'CG White Greek Cypriot'<br>'CH White Turkish'<br>'CJ White Turkish Cypriot'<br>'CK White Italian'<br>'CL White Irish Traveller'<br>'CM White Traveller'<br>'CN White Gypsy/Romany'<br>'CP White Polish'<br>'CQ White ex-USSR'<br>'CU White Croatian'<br>'CV White Serbian'<br>'CW White Other Ex-Yugoslav'<br>'CY White Other European'
**2 Mixed**		'CX White Mixed'<br>'D Mixed - White & Black Caribbean'<br>'E Mixed - White & Black African'<br>'F Mixed - White & Asian'<br>'G Mixed - Any other mixed background'<br>'GA Mixed - Black & Asian'<br>'GB Mixed - Black & Chinese'<br>'GC Mixed - Black & White'<br>'GD Mixed - Chinese & White'<br>'GE Mixed - Asian & Chinese'<br>'GF Mixed - Other/Unspecified'
**3 Asian**		'H Asian or Asian British - Indian'<br>'J Asian or Asian British - Pakistani'<br>'K Asian or Asian British - Bangladeshi'<br>'L Asian or Asian British - Any other Asian background'<br>'LA Asian Mixed'<br>'LB Asian Punjabi'<br>'LC Asian Kashmiri'<br>'LD Asian East African'<br>'LE Asian Sri Lankan'<br>'LF Asian Tamil'<br>'LG Asian Sinhalese'<br>'LH Asian British'<br>'LK Asian Unspecified'<br>'R Chinese'<br>'SA Vietnamese'<br>'SB Japanese'<br>'SC Filipino'<br>'SD Malaysian'
**4 Black**		'M Black or Black British - Caribbean'<br>'N Black or Black British - African'<br>'P Black or Black British - Any other Black background'<br>'PA Black Somali'<br>'PB Black Mixed'<br>'PC Black Nigerian'<br>'PD Black British'<br>'PE Black Unspecified'
**5 Other**		'SE Other Specified'

NER category	Data source code	Data source description
**1 White**		'A White - British'<br>'B White - Irish'<br>'C White - Any other White background'<br>'C2 White Northern Irish'<br>'C3 White Unspecified'<br>'CA White English'<br>'CB White Scottish'<br>'CC White Welsh'

NER category	Data source code	Data source description
		'CD White Cornish'<br>'CE White Cypriot (non specific)'<br>'CF White Greek'<br>'CG White Greek Cypriot'<br>'CH White Turkish'<br>'CJ White Turkish Cypriot'<br>'CK White Italian'<br>'CL White Irish Traveller'<br>'CM White Traveller'<br>'CN White Gypsy/Romany'<br>'CP White Polish'<br>'CQ White ex-USSR'<br>'CU White Croatian'<br>'CV White Serbian'<br>'CW White Other Ex-Yugoslav'<br>'CY White Other European'
**2 Mixed**		'CX White Mixed'<br>'D Mixed - White & Black Caribbean'<br>'E Mixed - White & Black African'<br>'F Mixed - White & Asian'<br>'G Mixed - Any other mixed background'<br>'GA Mixed - Black & Asian'<br>'GB Mixed - Black & Chinese'<br>'GC Mixed - Black & White'<br>'GD Mixed - Chinese & White'<br>'GE Mixed - Asian & Chinese'<br>'GF Mixed - Other/Unspecified'
**3 Indian**		'H Asian or Asian British – Indian’
**4 Pakistani**		'J Asian or Asian British - Pakistani'
**5 Bangladeshi**		'K Asian or Asian British - Bangladeshi'
**6 Chinese**		'R Chinese'
**7 Black Caribbean**		'M Black or Black British - Caribbean'
**8 Black African**		'N Black or Black British - African'<br>'PA Black Somali'<br>'PC Black Nigerian'
**9 Other**		'L Asian or Asian British - Any other Asian background'<br>'LA Asian Mixed'<br>'LB Asian Punjabi'<br>'LC Asian Kashmiri'<br>'LD Asian East African'<br>'LE Asian Sri Lankan'<br>'LF Asian Tamil'<br>'LG Asian Sinhalese'<br>'LH Asian British'<br>'LK Asian Unspecified'<br>'P Black or Black British - Any other Black background'<br>'PB Black Mixed'<br>'PD Black British'<br>'PE Black Unspecified'<br>'SA Vietnamese'<br>'SB Japanese'<br>'SC Filipino'<br>'SD Malaysian'<br>'SE Other Specified'

ICNC (ICNARC Intensive Care National Audit Research Centre)

ONS category	Data source code	Data source description
**1 White**	A<br>B<br>C	White, British<br>White, Irish<br>White, Any other
**2 Mixed**	D<br>E<br>F<br>G	Mixed, White and Black Caribbean<br>Mixed, White and Black African<br>Mixed, White and Asian<br>Mixed, Any other
**3 Asian**	H<br>J<br>K<br>L<br>R	Asian, Indian<br>Asian, Pakistani<br>Asian, Bangladeshi<br>Asian, Any other<br>Asian, Chinese
**4 Black**	M<br>N<br>P	Black, Caribbean<br>Black, African<br>Black, Any other
**5 Other**	S	Any other ethnic group

NER category	Data source code	Data source description
**1 White**	A<br>B<br>C	White, British<br>White, Irish<br>White, Any other
**2 Mixed**	D<br>E<br>F<br>G	Mixed, White and Black Caribbean<br>Mixed, White and Black African<br>Mixed, White and Asian<br>Mixed, Any other
**3 Indian**	H	Asian, Indian
**4 Pakistani**	J	Asian, Pakistani
**5 Bangladeshi**	K	Asian, Bangladeshi
**6 Chinese**	R	Asian, Chinese
**7 Black Caribbean**	M	Black, Caribbean
**8 Black African**	N	Black, African
**9 Other**	L<br>P<br>S	Asian, Any other<br>Black, Any other<br>Any other ethnic group

LACW (Looked After Children Wales)

ONS category	Data source code	Data source description
**1 White**	A1<br>A2<br>A3<br>WHTE	White British<br>White Irish<br>White Other<br>White
**2 Mixed**	B1<br>B2<br>B3<br>B4<br>MIXD	White and Black Caribbean<br>White and Black African<br>White and Asian<br>Mixed Background Other<br>Mixed ethnic groups
**3 Asian**	ASAB<br>C1<br>C2<br>C3<br>C4<br>E1	Asian or Asian British<br>Indian<br>Pakistani<br>Bangladeshi<br>Asian Other<br>Chinese
**4 Black**	BBAC<br>D1<br>D2<br>D3	Black; African; Caribbean or Black British<br>Caribbean<br>African<br>Black Background Other
**5 Other**	E2<br>OOTH	Any Other Ethnic Group<br>Other ethnic group

NER category	Data source code	Data source description
**1 White**	A1<br>A2<br>A3<br>WHTE	White British<br>White Irish<br>White Other<br>White
**2 Mixed**	B1<br>B2<br>B3<br>B4<br>MIXD	White and Black Caribbean<br>White and Black African<br>White and Asian<br>Mixed Background Other<br>Mixed ethnic groups
**3 Indian**	C1	Indian
**4 Pakistani**	C2	Pakistani
**5 Bangladeshi**	C3	Bangladeshi
**6 Chinese**	E1	Chinese
**7 Black Caribbean**	D1	Caribbean
**8 Black African**	D2	African
**9 Other**	ASAB<br>BBAC<br>C4<br>D3<br>E2<br>OOTH	Asian or Asian British<br>Black; African; Caribbean or Black British<br>Asian Other<br>Black Background Other<br>Any Other Ethnic Group<br>Other ethnic group

MIDS (Maternity Indicators DataSet)

ONS category	Data source code	Data source description
**1 White**	A<br><br>B	Any White Background, including Welsh, English, Scottish, Northern Irish, Irish, British<br>Gypsy or Irish Traveller
**2 Mixed**	D<br>E<br>F<br>G	White and Black Caribbean<br>White and Black African<br>White and Asian<br>Any other mixed background / multiple ethnic background
**3 Asian**	H<br>J<br>K<br>L<br>R	Indian<br>Pakistani<br>Bangladeshi<br>Any other Asian Background<br>Chinese
**4 Black**	M<br>N<br>P	Caribbean<br>African<br>Any other Black background
**5 Other**	S<br>T	Any other ethnic group<br>Arab

NER category	Data source code	Data source description
**1 White**	A<br><br>B	Any White Background, including Welsh, English, Scottish, Northern Irish, Irish, British<br>Gypsy or Irish Traveller
**2 Mixed**	D<br>E<br>F<br>G	White and Black Caribbean<br>White and Black African<br>White and Asian<br>Any other mixed background / multiple ethnic background
**3 Indian**	H	Indian
**4 Pakistani**	J	Pakistani
**5 Bangladeshi**	K	Bangladeshi
**6 Chinese**	R	Chinese
**7 Black Caribbean**	M	Caribbean
**8 Black African**	N	African
**9 Other**	L<br>P<br>S<br>T	Any other Asian Background<br>Any other Black background<br>Any other ethnic group<br>Arab

NCCH (National Community Child Health data)

ONS category	Data source code	Data source description
**1 White**	0<br>A<br><br>B	White<br>Any White Background, including Welsh, English, Scottish, Northern Irish, Irish, British<br>Gypsy or Irish Traveller
**2 Mixed**	D<br>E<br>F<br>G	White and Black Caribbean<br>White and Black African<br>White and Asian<br>Any other mixed background / multiple ethnic background
**3 Asian**	4<br>5<br>6<br>7<br>H<br>J<br>K<br>L<br>R	Indian<br>Pakistani<br>Bangladeshi<br>Chinese<br>Indian<br>Pakistani<br>Bangladeshi<br>Any other Asian Background<br>Chinese
**4 Black**	1<br>2<br>3<br>M<br>N<br>P	Black - Caribbean<br>Black - African<br>Black - Other<br>Caribbean<br>African<br>Any other Black background
**5 Other**	8<br>S<br>T	Any other ethnic group<br>Any other ethnic group<br>Arab

NER category	Data source code	Data source description
**1 White**	0<br>A<br><br>B	White<br>Any White Background, including Welsh, English, Scottish, Northern Irish, Irish, British<br>Gypsy or Irish Traveller
**2 Mixed**	D<br>E<br>F<br>G	White and Black Caribbean<br>White and Black African<br>White and Asian<br>Any other mixed background / multiple ethnic background
**3 Indian**	4<br>H	Indian<br>Indian
**4 Pakistani**	5<br>J	Pakistani<br>Pakistani
**5 Bangladeshi**	6<br>K	Bangladeshi<br>Bangladeshi
**6 Chinese**	7<br>R	Chinese<br>Chinese
**7 Black Caribbean**	1<br>M	Black - Caribbean<br>Caribbean
**8 Black African**	2<br>N	Black - African<br>African
**9 Other**	3<br>8<br>L<br>P<br>S<br>T	Black - Other<br>Any other ethnic group<br>Any other Asian Background<br>Any other Black background<br>Any other ethnic group<br>Arab

NHSO (NHS 111)

ONS category	Data source code	Data source description
**1 White**		'White - British'<br>'White - Any other White background'<br>'White - Irish'
**2 Mixed**		'Mixed - White and Black Caribbean'<br>'Mixed - White and Asian'<br>'Mixed - White and Black African'<br>'Mixed - Any other mixed background'
**3 Asian**		'Asian or Asian British - Indian'<br>'Asian or Asian British - Pakistani'<br>'Asian or Asian British - Bangladeshi'<br>'Other ethnic groups - Chinese'<br>'Asian or Asian British - Any other Asian background'
**4 Black**		'Black or Black British - Caribbean'<br>'Black or Black British - African'<br>'Black or Black British - Any other Black background'
**5 Other**		'Other ethnic groups - Any other ethnic group'

NER category	Data source code	Data source description
**1 White**		'White - British'<br>'White - Any other White background'<br>'White - Irish'
**2 Mixed**		'Mixed - White and Black Caribbean'<br>'Mixed - White and Asian'<br>'Mixed - White and Black African'<br>'Mixed - Any other mixed background'
**3 Indian**		'Asian or Asian British - Indian'
**4 Pakistani**		'Asian or Asian British - Pakistani'
**5 Bangladeshi**		'Asian or Asian British - Bangladeshi'
**6 Chinese**		'Other ethnic groups - Chinese'
**7 Black Caribbean**		'Black or Black British - Caribbean'
**8 Black African**		'Black or Black British - African'
**9 Other**		'Asian or Asian British - Any other Asian background'<br>'Black or Black British - Any other Black background'<br>'Other ethnic groups - Any other ethnic group'

NSWD (National Survey for Wales Data)

ONS category	Data source code	Data source description
**1 White**	1<br>2<br>3<br>4<br>19	White - Welsh/English/Scottish/Northern Irish/British<br>White - Irish<br>White - Gypsy or Irish Traveller<br>White – Other<br>White - Polish (SPONTANEOUS ONLY)
**2 Mixed**	5<br>6<br>7<br>8	Mixed - White and Black Caribbean<br>Mixed - White and Black African<br>Mixed - White and Asian<br>Mixed - Other
**3 Asian**	9<br>10<br>11<br>12<br>13	Asian - Indian<br>Asian - Pakistani<br>Asian - Bangladeshi<br>Asian - Chinese<br>Asian - Other
**4 Black**	14<br>15<br>16	Black - African<br>Black - Caribbean<br>Black - Other
**5 Other**	17<br>18	Other - Arab<br>Other - Any other ethnic group

NER category	Data source code	Data source description
**1 White**	1<br>2<br>3<br>4<br>19	White - Welsh/English/Scottish/Northern Irish/British<br>White - Irish<br>White - Gypsy or Irish Traveller<br>White – Other<br>White - Polish (SPONTANEOUS ONLY)
**2 Mixed**	5<br>6<br>7<br>8	Mixed - White and Black Caribbean<br>Mixed - White and Black African<br>Mixed - White and Asian<br>Mixed - Other
**3 Indian**	9	Asian - Indian
**4 Pakistani**	10	Asian - Pakistani
**5 Bangladeshi**	11	Asian - Bangladeshi
**6 Chinese**	12	Asian - Chinese
**7 Black Caribbean**	15	Black - Caribbean
**8 Black African**	14	Black - African
**9 Other**	13<br>16<br>17<br>18	Asian – Other<br>Black – Other<br>Other - Arab<br>Other - Any other ethnic group

OPRD (Out Patient Referrals Dataset)

ONS category	Data source code	Data source description
**1 White**	0<br>A<br><br>B	White<br>Any White Background, including Welsh, English, Scottish, Northern Irish, Irish, British<br>Gypsy or Irish Traveller
**2 Mixed**	D<br>E<br>F<br>G	White and Black Caribbean<br>White and Black African<br>White and Asian<br>Any other mixed background / multiple ethnic background
**3 Asian**	4<br>5<br>6<br>7<br>H<br>J<br>K<br>L<br>R	Indian<br>Pakistani<br>Bangladeshi<br>Chinese<br>Indian<br>Pakistani<br>Bangladeshi<br>Any other Asian Background<br>Chinese
**4 Black**	1<br>2<br>3<br>M<br>N<br>P	Black - Caribbean<br>Black - African<br>Black - Other<br>Caribbean<br>African<br>Any other Black background
**5 Other**	8<br>S<br>T	Any other ethnic group<br>Any other ethnic group<br>Arab

NER category	Data source code	Data source description
**1 White**	0<br>A<br><br>B	White<br>Any White Background, including Welsh, English, Scottish, Northern Irish, Irish, British<br>Gypsy or Irish Traveller
**2 Mixed**	D<br>E<br>F<br>G	White and Black Caribbean<br>White and Black African<br>White and Asian<br>Any other mixed background / multiple ethnic background
**3 Indian**	4<br>H	Indian<br>Indian
**4 Pakistani**	5<br>J	Pakistani<br>Pakistani
**5 Bangladeshi**	6<br>K	Bangladeshi<br>Bangladeshi
**6 Chinese**	7<br>R	Chinese<br>Chinese
**7 Black Caribbean**	1<br>M	Black - Caribbean<br>Caribbean
**8 Black African**	2<br>N	Black - African<br>African
**9 Other**	3<br>8<br>L<br>P<br>S<br>T	Black - Other<br>Any other ethnic group<br>Any other Asian Background<br>Any other Black background<br>Any other ethnic group<br>Arab

PEDW (Patient Episode Database for Wales)

ONS category	Data source code	Data source description
**1 White**	0<br>A<br><br>B	White<br>Any White Background, including Welsh, English, Scottish, Northern Irish, Irish, British<br>Gypsy or Irish Traveller
**2 Mixed**	D<br>E<br>F<br>G	White and Black Caribbean<br>White and Black African<br>White and Asian<br>Any other mixed background / multiple ethnic background
**3 Asian**	4<br>5<br>6<br>7<br>H<br>J<br>K<br>L<br>R	Indian<br>Pakistani<br>Bangladeshi<br>Chinese<br>Indian<br>Pakistani<br>Bangladeshi<br>Any other Asian Background<br>Chinese
**4 Black**	1<br>2<br>3<br>M<br>N<br>P	Black - Caribbean<br>Black - African<br>Black - Other<br>Caribbean<br>African<br>Any other Black background
**5 Other**	8<br>S<br>T	Any other ethnic group<br>Any other ethnic group<br>Arab

NER category	Data source code	Data source description
**1 White**	0<br>A<br><br>B	White<br>Any White Background, including Welsh, English, Scottish, Northern Irish, Irish, British<br>Gypsy or Irish Traveller
**2 Mixed**	D<br>E<br>F<br>G	White and Black Caribbean<br>White and Black African<br>White and Asian<br>Any other mixed background / multiple ethnic background
**3 Indian**	4<br>H	Indian<br>Indian
**4 Pakistani**	5<br>J	Pakistani<br>Pakistani
**5 Bangladeshi**	6<br>K	Bangladeshi<br>Bangladeshi
**6 Chinese**	7<br>R	Chinese<br>Chinese
**7 Black Caribbean**	1<br>M	Black - Caribbean<br>Caribbean
**8 Black African**	2<br>N	Black - African<br>African
**9 Other**	3<br>8<br>L<br>P<br>S<br>T	Black - Other<br>Any other ethnic group<br>Any other Asian Background<br>Any other Black background<br>Any other ethnic group<br>Arab

SACT (Systematic Anti Cancer Therapy)

ONS category	Data source code	Data source description
**1 White**		'Any White'
**2 Mixed**		'Mixed White and Asian'<br>'Mixed White and Black African'<br>'Mixed White and Black Caribbean'<br>'Other mixed'
**3 Asian**		'Bangladeshi'<br>'Chinese'<br>'Indian<br>'Other Asian',<br>'Pakistani'
**4 Black**		'Other Black'<br>'Black Caribbean'<br>'Black African'
**5 Other**		'Other'

NER category	Data source code	Data source description
**1 White**		'Any White'
**2 Mixed**		'Mixed White and Asian'<br>'Mixed White and Black African'<br>'Mixed White and Black Caribbean'<br>'Other mixed'
**3 Indian**		'Indian'
**4 Pakistani**		'Pakistani'
**5 Bangladeshi**		'Bangladeshi'
**6 Chinese**		'Chinese'
**7 Black Caribbean**		'Black Caribbean'
**8 Black African**		'Black African'
**9 Other**		'Other Asian'<br>'Other Black'<br>'Other'

SMDS (Substance Misuse DataSet)

ONS category	Data source code	Data source description
**1 White**	0<br>A<br><br>B	White<br>Any White Background, including Welsh, English, Scottish, Northern Irish, Irish, British<br>Gypsy or Irish Traveller
**2 Mixed**	D<br>E<br>F<br>G	White and Black Caribbean<br>White and Black African<br>White and Asian<br>Any other mixed background / multiple ethnic background
**3 Asian**	4<br>5<br>6<br>7<br>H<br>J<br>K<br>L<br>R	Indian<br>Pakistani<br>Bangladeshi<br>Chinese<br>Indian<br>Pakistani<br>Bangladeshi<br>Any other Asian Background<br>Chinese
**4 Black**	1<br>2<br>3<br>M<br>N<br>P	Black - Caribbean<br>Black - African<br>Black - Other<br>Caribbean<br>African<br>Any other Black background
**5 Other**	8<br>S<br>T	Any other ethnic group<br>Any other ethnic group<br>Arab

NER category	Data source code	Data source description
**1 White**	0<br>A<br><br>B	White<br>Any White Background, including Welsh, English, Scottish, Northern Irish, Irish, British<br>Gypsy or Irish Traveller
**2 Mixed**	D<br>E<br>F<br>G	White and Black Caribbean<br>White and Black African<br>White and Asian<br>Any other mixed background / multiple ethnic background
**3 Indian**	4<br>H	Indian<br>Indian
**4 Pakistani**	5<br>J	Pakistani<br>Pakistani
**5 Bangladeshi**	6<br>K	Bangladeshi<br>Bangladeshi
**6 Chinese**	7<br>R	Chinese<br>Chinese
**7 Black Caribbean**	1<br>M	Black - Caribbean<br>Caribbean
**8 Black African**	2<br>N	Black - African<br>African
**9 Other**	3<br>8<br>L<br>P<br>S<br>T	Black - Other<br>Any other ethnic group<br>Any other Asian Background<br>Any other Black background<br>Any other ethnic group<br>Arab

194 SWAC (School Workforce Annual Census)

ONS category	Data source code	Data source description
**1 White**	WALB	Albanian
	WBGR	British Gypsy / Gypsy Roma
	WBOS	Bosnian-Herzegovinian
	WBRI	White – British
	WBUL	Bulgarian
	WCRO	Croatian
	WCZE	Czech
	WEEU	White Eastern European
	WEUR	White European Other
	WFRE	French
	WGER	German
	WGRE	Greek / Greek Cypriot
	WGRO	Gypsy / Gypsy Roma from Other Countries
	WHUN	Hungarian
	WITA	Italian
	WITH	Traveller of Irish Heritage
	WIRT	Traveller
	WKOS	Kosovan
	WLAT	Latvian
	WLIT	Lithuanian
	WMAL	Maltese
	WMON	Montenegran
	WNAG	New Traveller
	WOBG	British Gypsy
	WOCC	Occupational Traveller
	WOER	EU Roma
	WOGR	Other Gypsy / Gypsy Roma
	WOOG	Gypsy from Other Countries
	WOOR	Roma from Other Countries
	WOTG	Other Gypsy
	WOTH	Any other White Background
	WOTR	Other Roma
	WOTT	Other Traveller
	WOTW	Other White
	WPOL	Polish
	WPOR	Portuguese
	WRMA	Romanian
	WROM	Gypsy / Gypsy Roma
	WRUS	Russian
	WSCA	Scandinavian
	WSER	Serbian
	WSPA	Spanish
	WSVK	Slovakian
	WSVN	Slovenian
	WTUR	Turkish / Turkish Cypriot
	WUKR	Ukrainian
	WWEU	White Western European
**2 Mixed**	MABL	Asian and Black
	MACH	Asian and Chinese
	MAOE	Asian and Any Other Ethnic Group
	MBCH	Black and Chinese
	MBOE	Black and Other Ethnic Group
	MCOE	Chinese and Any Other Ethnic Group
	MOTH	Any Other Mixed Background
	MOTM	Other Mixed Background
	MWAS	White and Asian
	MWBA	White and Black African
	MWBC	White and Black Caribbean
	MWCH	White and Chinese
	MWOE	White and Any Other Ethnic Group
**3 Asian**	AAFR	African Asian
	ABAN	Bangladeshi
	AIND	Indian
	AKAS	Kashmiri
	AMPK	Mirpuri Pakistani
	ANEP	Nepali
	AOTA	Other Asian
	AOTH	Any Other Asian Background
	APOK	Other Pakistani
	APKN	Pakistani
	ASNL	Sinhalese
	ASLT	Sri Lankan Tamil
	CHKC	Hong Kong Chinese

ONS category	Data source code	Data source description
	CHNE	Chinese or Chinese British
	CMAL	Malaysian Chinese
	CSNG	Singaporean Chinese
	CTWN	Taiwanese
	OFIL	Filipino
	OJPN	Japanese
	OKOR	Korean
	OMAL	Malay
	OOCH	Other Chinese
**4 Black**	BAFR	African
	BAOF	Other Black African
	BCRB	Caribbean
	BERI	Eritrean
	BEUR	Black European
	BGHA	Ghanaian
	BNAM	Black North American
	BOTB	Other Black
	BOTH	Any other Black background
	BNGN	Nigerian
	BSLN	Sierra Leonian
	BSOM	Somali
	BSUD	Sudanese
**5 Other**	OAFG	Afghanistani
	OARA	Arab
	OEGY	Egyptian
	OIRN	Iranian
	OIRQ	Iraqi
	OKRD	Kurdish
	OLAM	Latin / South / Central American
	OLEB	Lebanese
	OLIB	Libyan
	OMRC	Moroccan
	OOEG	Other ethnic group
	OOTH	Any other ethnic background
	OPOL	Polynesian
	OSYR	Syrian
	OTHA	Thai
	OSAU	Saudi Arabian
	OVIE	Vietnamese
	OYEM	Yemeni

NER category	Data source code	Data source description
**1 White**	WALB	Albanian
	WBGR	British Gypsy / Gypsy Roma
	WBOS	Bosnian-Herzegovinian
	WBRI	White – British
	WBUL	Bulgarian
	WCRO	Croatian
	WCZE	Czech
	WEEU	White Eastern European
	WEUR	White European Other
	WFRE	French
	WGER	German
	WGRE	Greek / Greek Cypriot
	WGRO	Gypsy / Gypsy Roma from Other Countries
	WHUN	Hungarian
	WITA	Italian
	WITH	Traveller of Irish Heritage
	WIRT	Traveller
	WKOS	Kosovan
	WLAT	Latvian
	WLIT	Lithuanian
	WMAL	Maltese
	WMON	Montenegran
	WNAG	New Traveller
	WOBG	British Gypsy
	WOCC	Occupational Traveller
	WOER	EU Roma
	WOGR	Other Gypsy / Gypsy Roma
	WOOG	Gypsy from Other Countries
	WOOR	Roma from Other Countries
	WOTG	Other Gypsy
	WOTH	Any other White Background

NER category	Data source code	Data source description
	WOTR	Other Roma
	WOTT	Other Traveller
	WOTW	Other White
	WPOL	Polish
	WPOR	Portuguese
	WRMA	Romanian
	WROM	Gypsy / Gypsy Roma
	WRUS	Russian
	WSCA	Scandinavian
	WSER	Serbian
	WSPA	Spanish
	WSVK	Slovakian
	WSVN	Slovenian
	WTUR	Turkish / Turkish Cypriot
	WUKR	Ukrainian
	WWEU	White Western European
**2 Mixed**	MABL	Asian and Black
	MACH	Asian and Chinese
	MAOE	Asian and Any Other Ethnic Group
	MBCH	Black and Chinese
	MBOE	Black and Other Ethnic Group
	MCOE	Chinese and Any Other Ethnic Group
	MOTH	Any Other Mixed Background
	MOTM	Other Mixed Background
	MWAS	White and Asian
	MWBA	White and Black African
	MWBC	White and Black Caribbean
	MWCH	White and Chinese
	MWOE	White and Any Other Ethnic Group
**3 Indian**	AIND	Indian
**4 Pakistani**	AMPK	Mirpuri Pakistani
	AOPK	Other Pakistani
	APKN	Pakistani
**5 Bangladeshi**	ABAN	Bangladeshi
**6 Chinese**	CHKC	Hong Kong Chinese
	CHNE	Chinese or Chinese British
	CMAL	Malaysian Chinese
	CSNG	Singaporean Chinese
	CTWN	Taiwanese
	OOCH	Other Chinese
**7 Black Caribbean**	BCRB	Caribbean
**8 Black African**	BAFR	African
	BAOF	Other Black African
	BERI	Eritrean
	BGHA	Ghanaian
	BNGN	Nigerian
	BSLN	Sierra Leonian
	BSOM	Somali
	BSUD	Sudanese
**9 Other**	AAFR	African Asian
	AKAS	Kashmiri
	ANEP	Nepali
	AOTA	Other Asian
	AOTH	Any Other Asian Background
	ASNL	Sinhalese
	ASLT	Sri Lankan Tamil
	BEUR	Black European
	BNAM	Black North American
	BOTB	Other Black
	OAFG	Afghanistani
	OARA	Arab
	OFIL	Filipino
	OIRN	Iranian
	OIRQ	Iraqi
	OJPN	Japanese
	OKOR	Korean
	OKRD	Kurdish
	OLAM	Latin / South / Central American
	OLEB	Lebanese
	OLIB	Libyan
	OMAL	Malay
	OOEG	Other ethnic group
	OOTH	Any other ethnic background

NER category	Data source code	Data source description
	OPOL	Polynesian
	OSAU	Saudi Arabian
	OSYR	Syrian
	OTHA	Thai
	OVIE	Vietnamese
	OYEM	Yemeni

WASD (Welsh Ambulance Service Dataset)

ONS category	Data source code	Data source description
**1 White**	1	British
	2	Irish
	3	Any other white background
**2 Mixed**	4	White and black Caribbean
	5	White and black African
	6	White and Asian
	7	Any other mixed background
**3 Asian**	8	Indian
	9	Pakistani
	10	Bangladeshi
	11	Any other Asian background
	15	Chinese
**4 Black**	12	Caribbean
	13	African
	14	Any other Black background
**5 Other**	16	Any other ethnic group

NER category	Data source code	Data source description
**1 White**	1	British
	2	Irish
	3	Any other white background
**2 Mixed**	4	White and black Caribbean
	5	White and black African
	6	White and Asian
	7	Any other mixed background
**3 Indian**	8	Indian
**4 Pakistani**	9	Pakistani
**5 Bangladeshi**	10	Bangladeshi
**6 Chinese**	15	Chinese
**7 Black Caribbean**	12	Caribbean
**8 Black African**	13	Africa
**9 Other**	11	Any other Asian background
	14	Any other Black background
	16	Any other ethnic group

WLGP (Wales Longitudinal General Practice)

ONS category	Data source code	Data source description
**1 White**	'9i0..'	British or mixed British - ethnic category 2001 census
	'9i00.'	White British - ethnic category 2001 census
	'9i1..'	Irish - ethnic category 2001 census
	'9i10.'	White Irish - ethnic category 2001 census
	'9i2..'	Other White background - ethnic category 2001 census
	'9i20.'	English - ethnic category 2001 census
	'9i21.'	Scottish - ethnic category 2001 census
	'9i22.'	Welsh - ethnic category 2001 census
	'9i23.'	Cornish - ethnic category 2001 census
	'9i24.'	Northern Irish - ethnic category 2001 census
	'9i25.'	Ulster Scots - ethnic category 2001 census
	'9i26.'	Cypriot (part not stated) - ethnic category 2001 census
	'9i27.'	Greek - ethnic category 2001 census
	'9i28.'	Greek Cypriot - ethnic category 2001 census
	'9i29.'	Turkish - ethnic category 2001 census
	'9i2A.'	Turkish Cypriot - ethnic category 2001 census
	'9i2B.'	Italian - ethnic category 2001 census
	'9i2C.'	Irish Traveller - ethnic category 2001 census
	'9i2D.'	Traveller - ethnic category 2001 census
	'9i2E.'	Gypsy/Romany - ethnic category 2001 census
	'9i2F.'	Polish - ethnic category 2001 census
	'9i2G.'	Baltic Estonian/Latvian/Lithuanian - ethn categ 2001 census
	'9i2H.'	Commonwealth (Russian) Indep States - ethn categ 2001 census
	'9i2J.'	Kosovan - ethnic category 2001 census
	'9i2K.'	Albanian - ethnic category 2001 census
	'9i2L.'	Bosnian - ethnic category 2001 census
	'9i2M.'	Croatian - ethnic category 2001 census
	'9i2N.'	Serbian - ethnic category 2001 census
	'9i2P.'	Other republics former Yugoslavia - ethnic categ 2001 census
	'9i2Q.'	Mixed Irish and other White - ethnic category 2001 census
	'9i2R.'	Oth White European/Euro unsp/Mixed Euro 2001 census
	'9i2S.'	Other mixed White - ethnic category 2001 census
	'9i2T.'	Other White or White unspecified ethnic category 2001 census

ONS category	Data source code	Data source description
	'9S1..'	White
	'9S10.'	White British
	'9S11.'	White Irish
	'9S12.'	Other white ethnic group
	'9S13.'	White Scottish
	'9S14.'	Other white British ethnic group
	'9SA9.'	Irish (NMO)
	'9SAA.'	Greek/Greek Cypriot (NMO)
	'9SAB.'	Turkish/Turkish Cypriot (NMO)
	'9SAC.'	Other European (NMO)
	'9SI..'	Irish traveller
	'9t00.'	White:Eng/Welsh/Scot/NI/Brit - England and Wales 2011 census
	'9t01.'	White: Irish - England and Wales ethnic category 2011 census
	'9t02.'	White: Gypsy/Irish Traveller - Eng+Wales eth cat 2011 census
	'9t03.'	White: other White backgrd- Eng+Wales ethnic cat 2011 census
	'9t10.'	White - Northern Ireland ethnic category 2011 census
	'9t11.'	Irish Traveller - Northern Ireland ethnic cat 2011 census
	'9t20.'	White: Scottish - Scotland ethnic category 2011 census
	'9t21.'	White: other British - Scotland ethnic category 2011 census
	'9t22.'	White: Irish - Scotland ethnic category 2011 census
	'9t24.'	White: Polish - Scotland ethnic category 2011 census
	'9t25.'	White: other White ethnic grp- Scotland ethnic cat 2011 cens
**2 Mixed**	'9i3..'	White and Black Caribbean - ethnic category 2001 census
	'9i4..'	White and Black African - ethnic category 2001 census
	'9i5..'	White and Asian - ethnic category 2001 census
	'9i6..'	Other Mixed background - ethnic category 2001 census
	'9i60.'	Black and Asian - ethnic category 2001 census
	'9i61.'	Black and Chinese - ethnic category 2001 census
	'9i62.'	Black and White - ethnic category 2001 census
	'9i63.'	Chinese and White - ethnic category 2001 census
	'9i65.'	Other Mixed or Mixed unspecified ethnic category 2001 census
	'9iA7.'	Caribbean Asian - ethnic category 2001 census
	'9S43.'	Black N African/Arab/Iranian
	'9S46.'	Black Indian sub-continent
	'9S47.'	Black - other Asian
	'9S48.'	Black Black - other
	'9S5..'	Black - other, mixed
	'9S51.'	Other Black - Black/White orig
	'9S52.'	Other Black - Black/Asian orig
	'9SA4.'	N African Arab/Iranian (NMO)
	'9SA5.'	Other African countries (NMO)
	'9SA6.'	E Afric Asian/Indo-Carib (NMO)
	'9SB..'	Other ethnic, mixed origin
	'9SB1.'	Other ethnic, Black/White orig
	'9SB2.'	Other ethnic, Asian/White orig
	'9SB3.'	Other ethnic, mixed white orig
	'9SB4.'	Other ethnic, other mixed orig
	'9SB5.'	Black Caribbean and White
	'9SB6.'	Black African and White
	'9t04.'	Mixed: White+Black Caribbean - Eng+Wales eth cat 2011 census
	'9t05.'	Mixed: White+Black African - Eng+Wales eth cat 2011 census
	'9t06.'	Mixed: White+Asian - Eng+Wales ethnic category 2011 census
	'9t07.'	Mixed: other Mixed/multiple backgrd - Eng+Wales 2011 census
	'9t12.'	Mixed: White and Black Caribbean - NI ethnic cat 2011 census
	'9t13.'	Mixed: White and Black African - NI ethnic cat 2011 census
	'9t14.'	Mixed: White and Asian - NI ethnic category 2011 census
	'9t15.'	Mixed: other Mixed/multiple ethnic backgrd - NI 2011 census
	'9t26.'	Mixed/multiple ethnic grps: any- Scot ethnic cat 2011 census
**3 Asian**	'9i64.'	Asian and Chinese - ethnic category 2001 census
	'9i7..'	Indian or British Indian - ethnic category 2001 census
	'9i8..'	Pakistani or British Pakistani - ethnic category 2001 census
	'9i9..'	Bangladeshi or British Bangladeshi - ethn categ 2001 census
	'9iA..'	Other Asian background - ethnic category 2001 census
	'9iA1.'	Punjabi - ethnic category 2001 census
	'9iA2.'	Kashmiri - ethnic category 2001 census
	'9iA3.'	East African Asian - ethnic category 2001 census
	'9iA4.'	Sri Lankan - ethnic category 2001 census
	'9iA5.'	Tamil - ethnic category 2001 census
	'9iA6.'	Sinhalese - ethnic category 2001 census
	'9iA8.'	British Asian - ethnic category 2001 census
	'9iA9.'	Mixed Asian - ethnic category 2001 census
	'9iAA.'	Other Asian or Asian unspecified ethnic category 2001 census
	'9iE..'	Chinese - ethnic category 2001 census
	'9iF0.'	Vietnamese - ethnic category 2001 census

ONS category	Data source code	Data source description
	'9iF1.'	Japanese - ethnic category 2001 census
	'9iF2.'	Filipino - ethnic category 2001 census
	'9iF3.'	Malaysian - ethnic category 2001 census
	'9S6..'	Indian
	'9S7..'	Pakistani
	'9S8..'	Bangladeshi
	'9S9..'	Chinese
	'9SA7.'	Indian sub-continent (NMO)
	'9SA8.'	Other Asian (NMO)
	'9SC..'	Vietnamese
	'9SH..'	Other Asian ethnic group
	'9t08.'	Asian/Asian Brit: Indian - Eng+Wales ethnic cat 2011 census
	'9t09.'	Asian/Asian British:Pakistani- Eng+Wales eth cat 2011 census
	'9t0A.'	Asian/Asian Brit: Bangladeshi- Eng+Wales eth cat 2011 census
	'9t0B.'	Asian/Asian Brit: Chinese - Eng+Wales ethnic cat 2011 census
	'9t0C.'	Asian/Asian Brit: other Asian- Eng+Wales eth cat 2011 census
	'9t16.'	Asian or Asian British: Indian - NI ethnic cat 2011 census
	'9t17.'	Asian/Asian British: Pakistani - NI ethnic cat 2011 census
	'9t18.'	Asian/Asian British: Bangladeshi - NI ethnic cat 2011 census
	'9t19.'	Asian/Asian British: Chinese - NI ethnic cat 2011 census
	'9t1A.'	Asian/Asian British: other Asian - NI ethnic cat 2011 census
	'9t27.'	Asian: Pakistani/Pakistani Scot/Pakistani Brit- Scot 2011
	'9t28.'	Asian: Indian, Indian Scot/Indian Brit- Scotland 2011 census
	'9t29.'	Bangladeshi, Bangladeshi Scot or Bangladeshi Brit- Scot 2011
	'9t2A.'	Asian: Chinese - Scotland ethnic category 2011 census
	'9t2B.'	Asian: other Asian group - Scotland ethnic cat 2011 census
**4 Black**	'9iB..'	Caribbean - ethnic category 2001 census
	'9iC..'	African - ethnic category 2001 census
	'9iD..'	Other Black background - ethnic category 2001 census
	'9iD0.'	Somali - ethnic category 2001 census
	'9iD1.'	Nigerian - ethnic category 2001 census
	'9iD2.'	Black British - ethnic category 2001 census
	'9iD3.'	Mixed Black - ethnic category 2001 census
	'9iD4.'	Other Black or Black unspecified ethnic category 2001 census
	'9iFA.'	North African - ethnic category 2001 census
	'9S2..'	Black Caribbean
	'9S3..'	Black African
	'9S4..'	Black, other, non-mixed origin
	'9S41.'	Black British
	'9S42.'	Black Caribbean/W.I./Guyana
	'9S44.'	Black - other African country
	'9S45.'	Black E Afric Asia/Indo-Caribb
	'9SA3.'	Caribbean I./W.I./Guyana (NMO)
	'9SG..'	Other black ethnic group
	'9t0D.'	Black/African/Carib/Black Brit: African- Eng+Wales 2011 cens
	'9t0E.'	Black/African/Caribbn/Black Brit: Caribbean - Eng+Wales 2011
	'9t0F.'	Black/Afr/Carib/Black Brit: other Black- Eng+Wales 2011 cens
	'9t1B.'	Black/Afri/Carib/Black Brit: African- NI eth cat 2011 census
	'9t1C.'	Black/Afri/Carib/Black Brit: Caribbean- NI eth cat 2011 cens
	'9t1D.'	Black/Afri/Carib/Black Brit: other - NI eth cat 2011 census
	'9t2C.'	African: African/African Scot/African Brit - Scotland 2011
	'9t2D.'	African: any other African - Scotland ethnic cat 2011 census
	'9t2E.'	Carib/Black: Caribbean/Carib Scot/Carib Brit- Scotland 2011
	'9t2F.'	Carib/Black: Black/Black Scot/Black Brit- Scotland 2011 cens
**5 Other**	'9iF..'	Other - ethnic category 2001 census
	'9iF4.'	Buddhist - ethnic category 2001 census
	'9iF5.'	Hindu - ethnic category 2001 census
	'9iF6.'	Jewish - ethnic category 2001 census
	'9iF7.'	Muslim - ethnic category 2001 census
	'9iF8.'	Sikh - ethnic category 2001 census
	'9iF9.'	Arab - ethnic category 2001 census
	'9iFB.'	Mid East (excl Israeli, Iranian & Arab) - eth cat 2001 cens
	'9iFC.'	Israeli - ethnic category 2001 census
	'9iFD.'	Iranian - ethnic category 2001 census
	'9iFE.'	Kurdish - ethnic category 2001 census
	'9iFF.'	Moroccan - ethnic category 2001 census
	'9iFG.'	Latin American - ethnic category 2001 census
	'9iFH.'	South and Central American - ethnic category 2001 census
	'9iFJ.'	Mauritian/Seychellois/Maldivian/St Helena eth cat 2001census
	'9iFK.'	Any other group - ethnic category 2001 census
	'9SA..'	Other ethnic non-mixed (NMO)
	'9SAD.'	Other ethnic NEC (NMO)
	'9SJ..'	Other ethnic group
	'9t0G.'	Other ethnic group: Arab - Eng+Wales ethnic cat 2011 census

ONS category	Data source code	Data source description
	'9t0H.'	Other ethnic: any other grp - Eng+Wales eth cat 2011 census
	'9t1E.'	Other ethnic group: Arab - NI ethnic category 2011 census
	'9t1F.'	Other ethnic group: any other grp- NI ethnic cat 2011 census
	'9t2H.'	Other ethnic grp: Arab/Arab Scot/Arab British- Scotland 2011
	'9t2J.'	Other ethnic grp: any other ethnic grp- Scotland 2011 census

202

NER category	Data source code	Data source description
**1 White**	'9i0..'	British or mixed British - ethnic category 2001 census
	'9i00.'	White British - ethnic category 2001 census
	'9i1..'	Irish - ethnic category 2001 census
	'9i10.'	White Irish - ethnic category 2001 census
	'9i2..'	Other White background - ethnic category 2001 census
	'9i20.'	English - ethnic category 2001 census
	'9i21.'	Scottish - ethnic category 2001 census
	'9i22.'	Welsh - ethnic category 2001 census
	'9i23.'	Cornish - ethnic category 2001 census
	'9i24.'	Northern Irish - ethnic category 2001 census
	'9i25.'	Ulster Scots - ethnic category 2001 census
	'9i26.'	Cypriot (part not stated) - ethnic category 2001 census
	'9i27.'	Greek - ethnic category 2001 census
	'9i28.'	Greek Cypriot - ethnic category 2001 census
	'9i29.'	Turkish - ethnic category 2001 census
	'9i2A.'	Turkish Cypriot - ethnic category 2001 census
	'9i2B.'	Italian - ethnic category 2001 census
	'9i2C.'	Irish Traveller - ethnic category 2001 census
	'9i2D.'	Traveller - ethnic category 2001 census
	'9i2E.'	Gypsy/Romany - ethnic category 2001 census
	'9i2F.'	Polish - ethnic category 2001 census
	'9i2G.'	Baltic Estonian/Latvian/Lithuanian - ethn categ 2001 census
	'9i2H.'	Commonwealth (Russian) Indep States - ethn categ 2001 census
	'9i2J.'	Kosovan - ethnic category 2001 census
	'9i2K.'	Albanian - ethnic category 2001 census
	'9i2L.'	Bosnian - ethnic category 2001 census
	'9i2M.'	Croatian - ethnic category 2001 census
	'9i2N.'	Serbian - ethnic category 2001 census
	'9i2P.'	Other republics former Yugoslavia - ethnic categ 2001 census
	'9i2Q.'	Mixed Irish and other White - ethnic category 2001 census
	'9i2R.'	Oth White European/Euro unsp/Mixed Euro 2001 census
	'9i2S.'	Other mixed White - ethnic category 2001 census
	'9i2T.'	Other White or White unspecified ethnic category 2001 census
	'9S1..'	White
	'9S10.'	White British
	'9S11.'	White Irish
	'9S12.'	Other white ethnic group
	'9S13.'	White Scottish
	'9S14.'	Other white British ethnic group
	'9SA9.'	Irish (NMO)
	'9SAA.'	Greek/Greek Cypriot (NMO)
	'9SAB.'	Turkish/Turkish Cypriot (NMO)
	'9SAC.'	Other European (NMO)
	'9SI..'	Irish traveller
	'9t00.'	White:Eng/Welsh/Scot/NI/Brit - England and Wales 2011 census
	'9t01.'	White: Irish - England and Wales ethnic category 2011 census
	'9t02.'	White: Gypsy/Irish Traveller - Eng+Wales eth cat 2011 census
	'9t03.'	White: other White backgrd- Eng+Wales ethnic cat 2011 census
	'9t10.'	White - Northern Ireland ethnic category 2011 census
	'9t11.'	Irish Traveller - Northern Ireland ethnic cat 2011 census
	'9t20.'	White: Scottish - Scotland ethnic category 2011 census
	'9t21.'	White: other British - Scotland ethnic category 2011 census
	'9t22.'	White: Irish - Scotland ethnic category 2011 census
	'9t24.'	White: Polish - Scotland ethnic category 2011 census
	'9t25.'	White: other White ethnic grp- Scotland ethnic cat 2011 cens
**2 Mixed**	'9i3..'	White and Black Caribbean - ethnic category 2001 census
	'9i4..'	White and Black African - ethnic category 2001 census
	'9i5..'	White and Asian - ethnic category 2001 census
	'9i6..'	Other Mixed background - ethnic category 2001 census
	'9i60.'	Black and Asian - ethnic category 2001 census
	'9i61.'	Black and Chinese - ethnic category 2001 census
	'9i62.'	Black and White - ethnic category 2001 census
	'9i63.'	Chinese and White - ethnic category 2001 census
	'9i65.'	Other Mixed or Mixed unspecified ethnic category 2001 census
	'9iA7.'	Caribbean Asian - ethnic category 2001 census
	'9S43.'	Black N African/Arab/Iranian

NER category	Data source code	Data source description
	'9S46.'<br>'9S47.'<br>'9S48.'<br>'9S5..'<br>'9S51.'<br>'9S52.'<br>'9SA4.'<br>'9SA5.'<br>'9SA6.'<br>'9SB..'<br>'9SB1.'<br>'9SB2.'<br>'9SB3.'<br>'9SB4.'<br>'9SB5.'<br>'9SB6.'<br>'9t04.'<br>'9t05.'<br>'9t06.'<br>'9t07.'<br>'9t12.'<br>'9t13.'<br>'9t14.'<br>'9t15.'<br>'9t26.'	Black Indian sub-continent<br>Black - other Asian<br>Black Black - other<br>Black - other, mixed<br>Other Black - Black/White orig<br>Other Black - Black/Asian orig<br>N African Arab/Iranian (NMO)<br>Other African countries (NMO)<br>E Afric Asian/Indo-Carib (NMO)<br>Other ethnic, mixed origin<br>Other ethnic, Black/White orig<br>Other ethnic, Asian/White orig<br>Other ethnic, mixed white orig<br>Other ethnic, other mixed orig<br>Black Caribbean and White<br>Black African and White<br>Mixed: White+Black Caribbean - Eng+Wales eth cat 2011 census<br>Mixed: White+Black African - Eng+Wales eth cat 2011 census<br>Mixed: White+Asian - Eng+Wales ethnic category 2011 census<br>Mixed: other Mixed/multiple backgrd - Eng+Wales 2011 census<br>Mixed: White and Black Caribbean - NI ethnic cat 2011 census<br>Mixed: White and Black African - NI ethnic cat 2011 census<br>Mixed: White and Asian - NI ethnic category 2011 census<br>Mixed: other Mixed/multiple ethnic backgrd - NI 2011 census<br>Mixed/multiple ethnic grps: any- Scot ethnic cat 2011 census
**3 Indian**	'9i7..'<br>'9S6..'<br>'9SA7.'<br>'9t08.'<br>'9t16.'<br>'9t28.'	Indian or British Indian - ethnic category 2001 census<br>Indian<br>Indian sub-continent (NMO)<br>Asian/Asian Brit: Indian - Eng+Wales ethnic cat 2011 census<br>Asian or Asian British: Indian - NI ethnic cat 2011 census<br>Asian: Indian, Indian Scot/Indian Brit- Scotland 2011 census
**4 Pakistani**	'9i8..'<br>'9S7..'<br>'9t09.'<br>'9t17.'<br>'9t27.'	Pakistani or British Pakistani - ethnic category 2001 census<br>Pakistani<br>Asian/Asian British:Pakistani- Eng+Wales eth cat 2011 census<br>Asian/Asian British: Pakistani - NI ethnic cat 2011 census<br>Asian: Pakistani/Pakistani Scot/Pakistani Brit- Scot 2011
**5 Bangladeshi**	'9i9..'<br>'9S8..'<br>'9t0A.'<br>'9t18.'<br>'9t29.'	Bangladeshi or British Bangladeshi - ethn categ 2001 census<br>Bangladeshi<br>Asian/Asian Brit: Bangladeshi- Eng+Wales eth cat 2011 census<br>Asian/Asian British: Bangladeshi - NI ethnic cat 2011 census<br>Bangladeshi, Bangladeshi Scot or Bangladeshi Brit- Scot 2011
**6 Chinese**	'9iE..'<br>'9S9..'<br>'9t0B.'<br>'9t19.'<br>'9t2A.'	Chinese - ethnic category 2001 census<br>Chinese<br>Asian/Asian Brit: Chinese - Eng+Wales ethnic cat 2011 census<br>Asian/Asian British: Chinese - NI ethnic cat 2011 census<br>Asian: Chinese - Scotland ethnic category 2011 census
**7 Black Caribbean**	'9iB..'<br>'9S2..'<br>'9S42.'<br>'9SA3.'<br>'9t2E.'<br>'9t2F.'	Caribbean - ethnic category 2001 census<br>Black Caribbean<br>Black Caribbean/W.I./Guyana<br>Caribbean I./W.I./Guyana (NMO)<br>Carib/Black: Caribbean/Carib Scot/Carib Brit- Scotland 2011<br>Carib/Black: Black/Black Scot/Black Brit- Scotland 2011 cens
**8 Black African**	'9iC..'<br>'9iD0.'<br>'9iD1.'<br>'9iFA.'<br>'9S3..'<br>'9S44.'<br>'9t2C.'<br>'9t2D.'	African - ethnic category 2001 census<br>Somali - ethnic category 2001 census<br>Nigerian - ethnic category 2001 census<br>North African - ethnic category 2001 census<br>Black African<br>Black - other African country<br>African: African/African Scot/African Brit - Scotland 2011<br>African: any other African - Scotland ethnic cat 2011 census
**9 Other**	'9i64.'<br>'9iA..'<br>'9iA1.'<br>'9iA2.'<br>'9iA3.'<br>'9iA4.'<br>'9iA5.'<br>'9iA6.'<br>'9iA8.'<br>'9iA9.'<br>'9iAA.'<br>'9iD..'<br>'9iD2.'<br>'9iD3.'	Asian and Chinese - ethnic category 2001 census<br>Other Asian background - ethnic category 2001 census<br>Punjabi - ethnic category 2001 census<br>Kashmiri - ethnic category 2001 census<br>East African Asian - ethnic category 2001 census<br>Sri Lankan - ethnic category 2001 census<br>Tamil - ethnic category 2001 census<br>Sinhalese - ethnic category 2001 census<br>British Asian - ethnic category 2001 census<br>Mixed Asian - ethnic category 2001 census<br>Other Asian or Asian unspecified ethnic category 2001 census<br>Other Black background - ethnic category 2001 census<br>Black British - ethnic category 2001 census<br>Mixed Black - ethnic category 2001 census

NER category	Data source code	Data source description
	'9iD4.'	Other Black or Black unspecified ethnic category 2001 census
	'9iF..'	Other - ethnic category 2001 census
	'9iF0.'	Vietnamese - ethnic category 2001 census
	'9iF1.'	Japanese - ethnic category 2001 census
	'9iF2.'	Filipino - ethnic category 2001 census
	'9iF3.'	Malaysian - ethnic category 2001 census
	'9iF4.'	Buddhist - ethnic category 2001 census
	'9iF5.'	Hindu - ethnic category 2001 census
	'9iF6.'	Jewish - ethnic category 2001 census
	'9iF7.'	Muslim - ethnic category 2001 census
	'9iF8.'	Sikh - ethnic category 2001 census
	'9iF9.'	Arab - ethnic category 2001 census
	'9iFB.'	Mid East (excl Israeli, Iranian & Arab) - eth cat 2001 cens
	'9iFC.'	Israeli - ethnic category 2001 census
	'9iFD.'	Iranian - ethnic category 2001 census
	'9iFE.'	Kurdish - ethnic category 2001 census
	'9iFF.'	Moroccan - ethnic category 2001 census
	'9iFG.'	Latin American - ethnic category 2001 census
	'9iFH.'	South and Central American - ethnic category 2001 census
	'9iFJ.'	Mauritian/Seychellois/Maldivian/St Helena eth cat 2001census
	'9iFK.'	Any other group - ethnic category 2001 census
	'9S4..'	Black, other, non-mixed origin
	'9S41.'	Black British
	'9S45.'	Black E Afric Asia/Indo-Caribb
	'9SA..'	Other ethnic non-mixed (NMO)
	'9SA8.'	Other Asian (NMO)
	'9SAD.'	Other ethnic NEC (NMO)
	'9SC..'	Vietnamese
	'9SG..'	Other black ethnic group
	'9SH..'	Other Asian ethnic group
	'9SJ..'	Other ethnic group
	'9t0C.'	Asian/Asian Brit: other Asian- Eng+Wales eth cat 2011 census
	'9t0D.'	Black/African/Carib/Black Brit: African- Eng+Wales 2011 cens
	'9t0E.'	Black/African/Caribbn/Black Brit: Caribbean - Eng+Wales 2011
	'9t0F.'	Black/Afr/Carib/Black Brit: other Black- Eng+Wales 2011 cens
	'9t0G.'	Other ethnic group: Arab - Eng+Wales ethnic cat 2011 census
	'9t0H.'	Other ethnic: any other grp - Eng+Wales eth cat 2011 census
	'9t1A.'	Asian/Asian British: other Asian - NI ethnic cat 2011 census
	'9t1B.'	Black/Afri/Carib/Black Brit: African- NI eth cat 2011 census
	'9t1C.'	Black/Afri/Carib/Black Brit: Caribbean- NI eth cat 2011 cens
	'9t1D.'	Black/Afri/Carib/Black Brit: other - NI eth cat 2011 census
	'9t2B.'	Asian: other Asian group - Scotland ethnic cat 2011 census
	'9t1E.'	Other ethnic group: Arab - NI ethnic category 2011 census
	'9t1F.'	Other ethnic group: any other grp- NI ethnic cat 2011 census
	'9t2H.'	Other ethnic grp: Arab/Arab Scot/Arab British- Scotland 2011
	'9t2J.'	Other ethnic grp: any other ethnic grp- Scotland 2011 census

Supplementary Table 3– meta-data of existing ethnicity RRDA tables

#	Column name	Type	Description
01	ALF_E	BIGINT	Anonymised Linkage Field (ALF)
02	ALF_E_C16	SMALLINT	ALF flag if in C19_COHORT16 (0 = NO / 1 = YES)
03	ALF_E_C20	SMALLINT	ALF flag if in C19_COHORT20 (0 = NO / 1 = YES)
04	ALF_E_C16_C20	SMALLINT	ALF flag if in C19_COHORT16 or C19_COHORT20 (0 = NO / 1 = YES)
05	ETHN_DATE_LATEST	DATE	Latest date method - Date of latest ethnicity recording
06	ETHN_EC_ONS_DATE_LATEST_CODE	SMALLINT	Latest date method code - Harmonised aggregate ethnicity - ONS 5 categories
07	ETHN_EC_ONS_DATE_LATEST_DESC	VARCHAR(15)	Latest date method description - Harmonised aggregate ethnicity - ONS 5 categories
08	ETHN_EC_NER_DATE_LATEST_CODE	SMALLINT	Latest date method code - Harmonised aggregate ethnicity - NER 9 categories
08	ETHN_EC_NER_DATE_LATEST_DESC	VARCHAR(15)	Latest date method description - Harmonised aggregate ethnicity - NER 9 categories
09	ETHN_EC_MIN_DATE_LATEST_CODE	SMALLINT	Latest date method code - Harmonised aggregate ethnicity - MIN 5 categories
10	ETHN_EC_MIN_DATE_LATEST_DESC	VARCHAR(15)	Latest date method description - Harmonised aggregate ethnicity - MIN 5 categories
11	ETHN_DATE_COUNTS	INTEGER	Number of ethnicity recordings in all data sources
12	ETHN_EC_ONS_MODE_CODE	SMALLINT	Mode (most common) method code - Harmonised aggregate ethnicity - ONS 5 categories
13	ETHN_EC_ONS_MODE_DESC	VARCHAR(15)	Mode (most common) method description - Harmonised aggregate ethnicity - ONS 5 categories
14	ETHN_EC_NER_MODE_CODE	SMALLINT	Mode (most common) method code - Harmonised aggregate ethnicity - NER 9 categories
15	ETHN_EC_NER_MODE_DESC	VARCHAR(15)	Mode (most common) method description - Harmonised aggregate ethnicity - NER 9 categories
16	ETHN_EC_MIN_MODE_CODE	SMALLINT	Mode (most common) method code - Harmonised aggregate ethnicity - MIN 5 categories
17	ETHN_EC_MIN_MODE_DESC	VARCHAR(15)	Mode (most common) method description - Harmonised aggregate ethnicity - MIN 5 categories
18	ETHN_EC_ONS_WMOD_CODE	SMALLINT	Weighted mode method code - Harmonised aggregate ethnicity - ONS 5 categories
19	ETHN_EC_ONS_WMOD_DESC	VARCHAR(15)	Weighted mode method description - Harmonised aggregate ethnicity - ONS 5 categories
20	ETHN_EC_NER_WMOD_CODE	SMALLINT	Weighted mode method code - Harmonised aggregate ethnicity - NER 9 categories
21	ETHN_EC_NER_WMOD_DESC	VARCHAR(15)	Weighted mode method description - Harmonised aggregate ethnicity - NER 9 categories
22	ETHN_EC_MIN_WMOD_CODE	SMALLINT	Weighted mode method code - Harmonised aggregate ethnicity - MIN 5 categories
23	ETHN_EC_MIN_WMOD_DESC	VARCHAR(15)	Weighted mode method description - Harmonised aggregate ethnicity - MIN 5 categories
24	ETHN_EC_ONS_COMP_LENGTH	INTEGER	Composite method - number of harmonised categories recorded - ONS 5 categories
25	ETHN_EC_ONS_COMP_ORIG_CODE	INTEGER	Composite method code - duplicate categories remain as duplicates - ONS 5 categories
26	ETHN_EC_ONS_COMP_ORIG_DESC	INTEGER	Composite method description - duplicate categories remain as duplicates - ONS 5 categories
27	ETHN_EC_ONS_COMP_MIXD_CODE	INTEGER	Composite method code - duplicate categories are assigned as MIXED - ONS 5 categories
28	ETHN_EC_ONS_COMP_MIXD_DESC	INTEGER	Composite method description - duplicate categories are assigned as MIXED - ONS 5 categories
29	ETHN_EC_ONS_COMP_NULL_CODE	INTEGER	Composite method code - duplicate categories are assigned as NULL - ONS 5 categories
30	ETHN_EC_ONS_COMP_NULL_DESC	INTEGER	Composite method description - duplicate categories are assigned as NULL - ONS 5 categories
31	ETHN_EC_NER_COMP_LENGTH	INTEGER	Composite method - number of harmonised categories recorded - NER 9 categories
32	ETHN_EC_NER_COMP_ORIG_CODE	INTEGER	Composite method code - duplicate categories remain as duplicates - NER 9 categories
33	ETHN_EC_NER_COMP_ORIG_DESC	INTEGER	Composite method description - duplicate categories remain as duplicates - NER 9 categories
34	ETHN_EC_NER_COMP_MIXD_CODE	INTEGER	Composite method code - duplicate categories are assigned as MIXED - NER 9 categories
35	ETHN_EC_NER_COMP_MIXD_DESC	INTEGER	Composite method description - duplicate categories are assigned as MIXED - NER 9 categories
36	ETHN_EC_NER_COMP_NULL_CODE	INTEGER	Composite method code - duplicate categories are assigned as NULL - NER 9 categories
37	ETHN_EC_NER_COMP_NULL_DESC	INTEGER	Composite method description - duplicate categories are assigned as NULL - NER 9 categories
38	ETHN_EC_MIN_COMP_LENGTH	INTEGER	Composite method - number of harmonised categories recorded - MIN 5 categories
32	ETHN_EC_MIN_COMP_ORIG_CODE	INTEGER	Composite method code - duplicate categories remain as duplicates -MIN 5 categories
33	ETHN_EC_MIN_COMP_ORIG_DESC	INTEGER	Composite method description - duplicate categories remain as duplicates - MIN 5 categories

34	ETHN_EC_MIN_COMP_MIXD_CODE	INTEGER	Composite method code - duplicate categories are assigned as MIXED - MIN 5 categories
35	ETHN_EC_MIN_COMP_MIXD_DESC	INTEGER	Composite method description - duplicate categories are assigned as MIXED - MIN 5 categories
36	ETHN_EC_MIN_COMP_NULL_CODE	INTEGER	Composite method code - duplicate categories are assigned as NULL - MIN 5 categories
37	ETHN_EC_MIN_COMP_NULL_DESC	INTEGER	Composite method description - duplicate categories are assigned as NULL - MIN 5 categories

RRDA_ETHN_PREP_DATE

#	Column name	Type	Description
01	ALF_E	BIGINT	Anonymised Linkage Field (ALF)
02	ALF_E_C16	SMALLINT	ALF flag if in C19_COHORT16 (0 = NO / 1 = YES)
03	ALF_E_C20	SMALLINT	ALF flag if in C19_COHORT20 (0 = NO / 1 = YES)
04	ALF_E_C16_C20	SMALLINT	ALF flag if in C19_COHORT16 or C19_COHORT20 (0 = NO / 1 = YES)
05	ETHN_DATA_SOURCE	CHAR(4)	Data source the ethnicity recording is from
06	ETHN_DATE	DATE	Date of ethnicity recording
07	ETHN_EC_ONS_CODE	SMALLINT	Harmonised aggregate ethnicity code - ONS 5 categories
08	ETHN_EC_ONS_DESC	VARCHAR(15)	Harmonised aggregate ethnicity description - ONS 5 categories
09	ETHN_EC_ONS_ORDER	SMALLINT	Ordered list (newest num 1) of all ethnicity recorded dates
10	ETHN_EC_NER_CODE	SMALLINT	Harmonised aggregate ethnicity code - NER 9 categories
11	ETHN_EC_NER_DESC	VARCHAR(15)	Harmonised aggregate ethnicity description - NER 9 categories
12	ETHN_EC_NER_ORDER	SMALLINT	Ordered list (newest num 1) of all ethnicity recorded dates
13	ETHN_EC_MIN_CODE	SMALLINT	Harmonised aggregate ethnicity code - MIN 5 categories
14	ETHN_EC_MIN_DESC	VARCHAR(15)	Harmonised aggregate ethnicity description - MIN 5 categories
15	ETHN_EC_MIN_ORDER	SMALLINT	Ordered list (newest num 1) of all ethnicity recorded dates
16	ETHN_DATE_LATEST	DATE	Latest date method - Date of latest ethnicity recording
17	ETHN_EC_ONS_DATE_LATEST_CODE	SMALLINT	Latest date method code - Harmonised aggregate ethnicity - ONS 5 categories
18	ETHN_EC_ONS_DATE_LATEST_DESC	VARCHAR(15)	Latest date method description - Harmonised aggregate ethnicity - ONS 5 categories
19	ETHN_EC_NER_DATE_LATEST_CODE	SMALLINT	Latest date method code - Harmonised aggregate ethnicity - NER 9 categories
20	ETHN_EC_NER_DATE_LATEST_DESC	VARCHAR(15)	Latest date method description - Harmonised aggregate ethnicity - NER 9 categories
21	ETHN_EC_MIN_DATE_LATEST_CODE	SMALLINT	Latest date method code - Harmonised aggregate ethnicity - MIN 5 categories
22	ETHN_EC_MIN_DATE_LATEST_DESC	VARCHAR(15)	Latest date method description - Harmonised aggregate ethnicity - MIN 5 categories
23	ETHN_DATE_COUNTS	INTEGER	Number of ethnicity recordings in all data sources

Supplementary Table 4– characteristics of the cohort based on ONS categorisation.

Characteristics	Latest Ethnic group N = 3,457,694 N (%)		Mode Ethnic group N = 3,457,694 N (%)		Weighted Mod Ethnic group N = 3,457,694 N (%)		Composite Ethnic group N = 3,457,694 N (%)	
**Age group**								
0-4	169,928	(5.0%)	169,928	(5.0%)	169,928	(5.0%)	169,928	(5.0%)
5-9	187,475	(5.5%)	187,475	(5.5%)	187,475	(5.5%)	187,475	(5.5%)
10-14	186,229	(5.5%)	186,229	(5.5%)	186,229	(5.5%)	186,229	(5.5%)
15-19	196,182	(5.8%)	196,182	(5.8%)	196,182	(5.8%)	196,182	(5.8%)
20-24	229,415	(6.7%)	229,415	(6.7%)	229,415	(6.7%)	229,415	(6.7%)
25-29	227,170	(6.7%)	227,170	(6.7%)	227,170	(6.7%)	227,170	(6.7%)
30-34	227,025	(6.7%)	227,025	(6.7%)	227,025	(6.7%)	227,025	(6.7%)
35-39	215,231	(6.3%)	215,231	(6.3%)	215,231	(6.3%)	215,231	(6.3%)
40-44	192,936	(5.7%)	192,936	(5.7%)	192,936	(5.7%)	192,936	(5.7%)
45-49	216,848	(6.4%)	216,848	(6.4%)	216,848	(6.4%)	216,848	(6.4%)
50-54	236,812	(7.0%)	236,812	(7.0%)	236,812	(7.0%)	236,812	(7.0%)
55-59	235,862	(6.9%)	235,862	(6.9%)	235,862	(6.9%)	235,862	(6.9%)
60-64	203,203	(6.0%)	203,203	(6.0%)	203,203	(6.0%)	203,203	(6.0%)
65-69	186,101	(5.5%)	186,101	(5.5%)	186,101	(5.5%)	186,101	(5.5%)
70-74	182,148	(5.4%)	182,148	(5.4%)	182,148	(5.4%)	182,148	(5.4%)
75-79	131,869	(3.9%)	131,869	(3.9%)	131,869	(3.9%)	131,869	(3.9%)
80-84	91,464	(2.7%)	91,464	(2.7%)	91,464	(2.7%)	91,464	(2.7%)
85+	86,322	(2.5%)	86,322	(2.5%)	86,322	(2.5%)	86,322	(2.5%)
**Sex**								
Male	1,728,015	(50.0%)	1,728,015	(50.0%)	1,728,015	(50.0%)	1,728,015	(50.0%)
Female	1,729,679	(50.0%)	1,729,679	(50.0%)	1,729,679	(50.0%)	1,729,679	(50.0%)
**WIMD 2019 quintiles**								
1-Most deprived	658,913	(21.0%)	658,913	(21.0%)	658,913	(21.0%)	658,913	(21.0%)
2	638,925	(20.0%)	638,925	(20.0%)	638,925	(20.0%)	638,925	(20.0%)
3	643,624	(20.0%)	643,624	(20.0%)	643,624	(20.0%)	643,624	(20.0%)
4	630,614	(20.0%)	630,614	(20.0%)	630,614	(20.0%)	630,614	(20.0%)
5-Least deprived	635,890	(20.0%)	635,890	(20.0%)	635,890	(20.0%)	635,890	(20.0%)
**UHB of residence**								
Aneurin Bevan	605,782	(19.0%)	605,782	(19.0%)	605,782	(19.0%)	605,782	(19.0%)
Betsi Cadwaladr	701,543	(22.0%)	701,543	(22.0%)	701,543	(22.0%)	701,543	(22.0%)
Cardiff and Vale	526,652	(16.0%)	526,652	(16.0%)	526,652	(16.0%)	526,652	(16.0%)
Cwm Taf Morgannwg	459,118	(14.0%)	459,118	(14.0%)	459,118	(14.0%)	459,118	(14.0%)
Hywel Dda	385,174	(12.0%)	385,174	(12.0%)	385,174	(12.0%)	385,174	(12.0%)
Powys	128,461	(4.0%)	128,461	(4.0%)	128,461	(4.0%)	128,461	(4.0%)
Swansea Bay	401,236	(13.0%)	401,236	(13.0%)	401,236	(13.0%)	401,236	(13.0%)
**Urban-rural category of residence**								
Urban city and town	2,179,684	(68.0%)	2,179,684	(68.0%)	2,179,684	(68.0%)	2,179,684	(68.0%)
Urban city and town in a sparse setting	59,602	(1.9%)	59,602	(1.9%)	59,602	(1.9%)	59,602	(1.9%)
Rural town and fringe	419,900	(13.0%)	419,900	(13.0%)	419,900	(13.0%)	419,900	(13.0%)
Rural village and dispersed	204,993	(6.4%)	204,993	(6.4%)	204,993	(6.4%)	204,993	(6.4%)
Rural village and dispersed in a sparse setting	225,307	(7.0%)	225,307	(7.0%)	225,307	(7.0%)	225,307	(7.0%)
Rural town and fringe in a sparse setting	118,480	(3.7%)	118,480	(3.7%)	118,480	(3.7%)	118,480	(3.7%)

Supplementary Table 5 – characteristics of the cohort based on NER categorisation.

Characteristics	Latest Ethnic group N = 3,457,694 N(%)		Mode Ethnic group N = 3,457,694 N(%)		Weighted Mode Ethnic group N = 3,457,694 N(%)		Composite Ethnic group N = 3,457,694 N(%)	
**Age group**								
0-4	169,928	(5.0%)	169,928	(5.0%)	169,928	(5.0%)	169,928	(5.0%)
5-9	187,475	(5.5%)	187,475	(5.5%)	187,475	(5.5%)	187,475	(5.5%)
10-14	186,229	(5.5%)	186,229	(5.5%)	186,229	(5.5%)	186,229	(5.5%)
15-19	196,182	(5.8%)	196,182	(5.8%)	196,182	(5.8%)	196,182	(5.8%)
20-24	229,415	(6.7%)	229,415	(6.7%)	229,415	(6.7%)	229,415	(6.7%)
25-29	227,170	(6.7%)	227,170	(6.7%)	227,170	(6.7%)	227,170	(6.7%)
30-34	227,025	(6.7%)	227,025	(6.7%)	227,025	(6.7%)	227,025	(6.7%)
35-39	215,231	(6.3%)	215,231	(6.3%)	215,231	(6.3%)	215,231	(6.3%)
40-44	192,936	(5.7%)	192,936	(5.7%)	192,936	(5.7%)	192,936	(5.7%)

45-49	216,848	(6.4%)	216,848	(6.4%)	216,848	(6.4%)	216,848	(6.4%)
50-54	236,812	(7.0%)	236,812	(7.0%)	236,812	(7.0%)	236,812	(7.0%)
55-59	235,862	(6.9%)	235,862	(6.9%)	235,862	(6.9%)	235,862	(6.9%)
60-64	203,203	(6.0%)	203,203	(6.0%)	203,203	(6.0%)	203,203	(6.0%)
65-69	186,101	(5.5%)	186,101	(5.5%)	186,101	(5.5%)	186,101	(5.5%)
70-74	182,148	(5.4%)	182,148	(5.4%)	182,148	(5.4%)	182,148	(5.4%)
75-79	131,869	(3.9%)	131,869	(3.9%)	131,869	(3.9%)	131,869	(3.9%)
80-84	91,464	(2.7%)	91,464	(2.7%)	91,464	(2.7%)	91,464	(2.7%)
85+	86,322	(2.5%)	86,322	(2.5%)	86,322	(2.5%)	86,322	(2.5%)
**Sex**								
Male	1,728,015	(50.0%)	1,728,015	(50.0%)	1,728,015	(50.0%)	1,728,015	(50.0%)
Female	1,729,679	(50.0%)	1,729,679	(50.0%)	1,729,679	(50.0%)	1,729,679	(50.0%)
**WIMD 2019 quintiles**								
1-Most deprived	658,913	(21.0%)	658,913	(21.0%)	658,913	(21.0%)	658,913	(21.0%)
2	638,925	(20.0%)	638,925	(20.0%)	638,925	(20.0%)	638,925	(20.0%)
3	643,624	(20.0%)	643,624	(20.0%)	643,624	(20.0%)	643,624	(20.0%)
4	630,614	(20.0%)	630,614	(20.0%)	630,614	(20.0%)	630,614	(20.0%)
5-Least deprived	635,890	(20.0%)	635,890	(20.0%)	635,890	(20.0%)	635,890	(20.0%)
**University Health Board (UHB)**								
Aneurin Bevan	605,782	(19.0%)	605,782	(19.0%)	605,782	(19.0%)	605,782	(19.0%)
Betsi Cadwaladr	701,543	(22.0%)	701,543	(22.0%)	701,543	(22.0%)	701,543	(22.0%)
Cardiff and Vale	526,652	(16.0%)	526,652	(16.0%)	526,652	(16.0%)	526,652	(16.0%)
Cwm Taf Morgannwg	459,118	(14.0%)	459,118	(14.0%)	459,118	(14.0%)	459,118	(14.0%)
Hywel Dda	385,174	(12.0%)	385,174	(12.0%)	385,174	(12.0%)	385,174	(12.0%)
Powys	128,461	(4.0%)	128,461	(4.0%)	128,461	(4.0%)	128,461	(4.0%)
Swansea Bay	401,236	(13.0%)	401,236	(13.0%)	401,236	(13.0%)	401,236	(13.0%)
**Urban-rural category of residence**								
Urban city and town	2,179,684	(68.0%)	2,179,684	(68.0%)	2,179,684	(68.0%)	2,179,684	(68.0%)
Urban city and town in a sparse setting	59,602	(1.9%)	59,602	(1.9%)	59,602	(1.9%)	59,602	(1.9%)
Rural town and fringe	419,900	(13.0%)	419,900	(13.0%)	419,900	(13.0%)	419,900	(13.0%)
Rural village and dispersed in a sparse setting	225,307	(7.0%)	225,307	(7.0%)	225,307	(7.0%)	225,307	(7.0%)
Rural village and dispersed	204,993	(6.4%)	204,993	(6.4%)	204,993	(6.4%)	204,993	(6.4%)
Rural town and fringe in a sparse setting	118,480	(3.7%)	118,480	(3.7%)	118,480	(3.7%)	118,480	(3.7%)
