# Supplementary material for: Exploring ethnicity dynamics in Wales: a longitudinal population-scale linked data study and development of a harmonised ethnicity spine
Source: BMJ Open. 2024 Aug 2;14(8):e077675. doi: 10.1136/bmjopen-2023-077675 (PMC11733787; doi:10.1136/bmjopen-2023-077675)
Supplement: Supplementary file 2 [file bmjopen-2023-077675.reviewer_comments.pdf]

PEER REVIEW HISTORY

BMJ Open publishes all reviews undertaken for accepted manuscripts. Reviewers are asked to complete a checklist review form (**http://bmjopen.bmj.com/site/about/resources/checklist.pdf**) and are provided with free text boxes to elaborate on their assessment. These free text comments are reproduced below.

ARTICLE DETAILS

**TITLE (PROVISIONAL)**	Exploring ethnicity dynamics in Wales: a longitudinal population-scale linked data study and development of a harmonised ethnicity-spine.
**AUTHORS**	Akbari, Ashley; Torabi, Fatemeh; Bedston, Stuart; Lowthian, Emily; Abbasizanjani, Hoda; Fry, Richard; Lyons, Jane; Owen, Rhiannon; Khunti, Kamlesh; Lyons, Ronan

VERSION 1 – REVIEW

**REVIEWER**	Battersby, Cheryl<br>Imperial College London, Neonatal Medicine
**REVIEW RETURNED**	03-Oct-2023

**GENERAL COMMENTS**	This is an excellent paper on a really important area. This paper demonstrates the benefits of including ethnic group information when understanding clinical outcomes at a population scale.<br><br>Ethnicity, whilst important and a focal point of many research studies, is recorded in many different ways in different databases and electronic health record systems. As an increasing number of EHR or databases exist, it is important to bring together data from all different sources in order to derive the most likely and accurate ethnicity of an individual.<br><br>The study aims to create a national ethnicity-spine based on all available ethnicity records in linkable anonymised HER and administrative data resources in the SAIL Welsh databank. SAIL has a renowned reputation nationally and internationally for his rich and comprehensive datasets and clinical governance processes in its safe haven.<br><br>The authors have compared different methods to derive ethnicity from multiple sources. These include Latest, Mode and Weighted-Mode approaches. They have described the different methods clearly in an easily understandable way in plain English:<br>The Latest method (most commonly used) uses the most recent point in time of their longitudinal records, so as to respect a possible change in self-identification.<br>The Mode and Weighted-mode take into account variation in reporting between data sources and also longitudinally to identify the most commonly reported ethnic group; this handles data error and variability to assign the most frequently reported ethnic group over the whole period of their research study period. The Composite method does not take into account temporal changes (as with the Latest method) or the most frequent ethnic group but assigns an ethnic group if unique across all data sources; or if two or more different ethnic groups are found then assigns the ethnic

	group as Mixed. It does not prioritise one record and ethnic group over another. The authors have a detailed discussion on the limitations of the composite method. They suggest that the composite approach is only appropriate in certain study design circumstances with a fixed point in time, no updates in data coverage and a need to retain the knowledge of individuals that declare to more than one ethnic group over time as a single row. They show that categorisation of individuals who declared more than one ethnicity over time into a single category can introduce major biases in the true distribution of ethnicity in the population.<br><br>Results:<br><br>The results demonstrated that the Latest, Mode and Weighted-Mode approaches all provided suitable categorisations for ethnic group data and the choice of ethnic group.<br>The Latest method provided a substantial agreement measure of ethnicity in the population when compared directly with the census, which is a promising improvement.<br><br>The distribution of the dominant group (White) is minimally affected based on the four different selection approaches. Across all other ethnic group categorisations, the Mixed group was most susceptible to variation in distribution depending on the selection approach used.<br><br>Discussion<br><br>Discussion is thoughtful and includes innovative ideas, such as incorporating self-reported ethnicity measurements with appropriate weightings applied to the data source contributions. The authors discuss the usability of the different methods and to consider the choice of harmonisation method given the findings from this study.<br><br>The paper is well written and mostly clear; I have suggestions for improvements to improve its structure and readability.<br><br>1) Aims/ clarify terms “categorisation” and “selection” and be consistent<br><br>“Here, we aimed to address the urgent need for improved harmonised ethnicity data considering two main factors, namely: categorisation and selection. We assessed the availability, record completeness and distribution of ethnicity records across EHRs and administrative data sources for two categorisation approaches and four approaches in assigning ethnic groups.”<br><br>- I think it is a bit confusing through the manuscript what are the categories and selection. I would suggest this is defined in this paragraph upfront.<br><br>I see this is explained further down: “two different categorisation methods: NER and ONS within each data source (Figure 2.A (15,25)- we refer to this stage as categorisation hereafter), and finally 3) serialising these records into a single row per individual using four different selection approaches: Composite, Latest,

	Mode, Weighted-Mode - to assign a single ethnicity to each individual (we refer to this stage as record selection hereafter)"<br><br>I think it needs to be clear that you mean NER and ONS ethnic categories and " selection" refer to the different data derivation processes.<br><br>2)<br><br>Discussion<br>I have very few suggestions for improvement. Only suggestion is that the discussion could be shortened and structured differently :<br><br>1) First (quite long) paragraph of discussion is extensively on the completeness, yet this is not stressed in the abstract aims – if the authors want the completeness to be a main outcome, can they please include this in the methods / aims? As second paragraph then goes onto the ethnicity spine.<br><br>2) Second paragraph-Again I think the terminology needs to be clear what categorisation and selection refer to, even if it is already clear in methods.<br><br>3) The paragraph "Our study demonstrates that the Latest, Mode and Weighted-Mode approaches all provide suitable categorisations for ethnic group data and the choice of ethnic group methodological approach chosen by any study down to their respective study design, with the standard choice from current studies across the UK being the Latest (Figures 3 & 4 - Table 3)."<br><br>This paragraph seems rather out of place and disjointed from the rest of the discussion all on its own. Can the authors please improve the flow – prob better incorporated into a paragraph higher up?<br><br>4) Suggest to add in a more generic closing/conclusion statement in discussion:<br><br>"In conclusion, our development of the population-scale ethnicity spine provides robust ethnicity measures to healthcare research in Wales and a template which can easily be deployed in other TREs in the UK and beyond"<br><br>5) there are a lot of supplementary files and it may be worthwhile to consider smaller font / tables<br><br>Minor<br>Typos<br>"Errornous" page 4 line 41/42 needs correcting please.

VERSION 1 – AUTHOR RESPONSE

Reviewer #1 - Dr. Cheryl Battersby, Imperial College London
This is an excellent paper on a really important area. This paper demonstrates the benefits of including ethnic group information when understanding clinical outcomes at a population scale.

Ethnicity, whilst important and a focal point of many research studies, is recorded in many different ways in different databases and electronic health record systems. As an increasing number of EHR or databases exist, it is important to bring together data from all different sources in order to derive the most likely and accurate ethnicity of an individual. The study aims to create a national ethnicity-spine based on all available ethnicity records in linkable anonymised HER and administrative data resources in the SAIL Welsh databank. SAIL has a renowned reputation nationally and internationally for his rich and comprehensive datasets and clinical governance processes in its safe haven. The authors have compared different methods to derive ethnicity from multiple sources. These include Latest, Mode and Weighted-Mode approaches. They have described the different methods clearly in an easily understandable way in plain English: The Latest method (most commonly used) uses the most recent point in time of their longitudinal records, so as to respect a possible change in self-identification.
The Mode and Weighted-mode take into account variation in reporting between data sources and also longitudinally to identify the most commonly reported ethnic group; this handles data error and variability to assign the most frequently reported ethnic group over the whole period of their research study period. The Composite method does not take into account temporal changes (as with the Latest method) or the most frequent ethnic group but assigns an ethnic group if unique across all data sources; or if two or more different ethnic groups are found then assigns the ethnic group as Mixed. It does not prioritise one record and ethnic group over another. The authors have a detailed discussion on the limitations of the composite method. They suggest that the composite approach is only appropriate in certain study design circumstances with a fixed point in time, no updates in data coverage and a need to retain the knowledge of individuals that declare to more than one ethnic group over time as a single row. They show that categorisation of individuals who declared more than one ethnicity over time into a single category can introduce major biases in the true distribution of ethnicity in the population.

Results:
The results demonstrated that the Latest, Mode and Weighted-Mode approaches all provided suitable categorisations for ethnic group data and the choice of ethnic group.
The Latest method provided a substantial agreement measure of ethnicity in the population when compared directly with the census, which is a promising improvement. The distribution of the dominant group (White) is minimally affected based on the four different selection approaches. Across all other ethnic group categorisations, the Mixed group was most susceptible to variation in distribution depending on the selection approach used.

Discussion
Discussion is thoughtful and includes innovative ideas, such as incorporating self-reported ethnicity measurements with appropriate weightings applied to the data source contributions. The authors discuss the usability of the different methods and to consider the choice of harmonisation method given the findings from this study.
The paper is well written and mostly clear; I have suggestions for improvements to improve its structure and readability.

1) Aims/ clarify terms “categorisation” and “selection” and be consistent.
“Here, we aimed to address the urgent need for improved harmonised ethnicity data considering two

main factors, namely: categorisation and selection. We assessed the availability, record completeness and distribution of ethnicity records across EHRs and administrative data sources for two categorisation approaches and four approaches in assigning ethnic groups."

Thank you for your constructive feedback; it helped us improve the consistency of the manuscript. The above paragraph is now reading as:
"Here, we aimed to improve harmonised ethnicity data. We assessed the availability, record completeness and distribution of ethnicity records across EHRs and administrative data sources. We compared four different methods for deriving ethnicity from multiple data sources, referred to as record selection approaches. These four selection approaches are namely: Latest, Mode, Weighted-Mode and Composite. "

- I think it is a bit confusing through the manuscript what are the categories and selection. I would suggest this is defined in this paragraph upfront.
I see this is explained further down: "two different categorisation methods: NER and ONS within each data source (Figure 2.A (15,25)- we refer to this stage as categorisation hereafter), and finally 3) serialising these records into a single row per individual using four different selection approaches: Composite, Latest, Mode, Weighted-Mode - to assign a single ethnicity to each individual (we refer to this stage as record selection hereafter)"
I think it needs to be clear that you mean NER and ONS ethnic categories and " selection" refer to the different data derivation processes.

We further tried to present the details on NER and ONS categorisation methods earlier in the text and ensure that these have been applied to all selection approaches. It now reads as follows:
"In addition to selection approaches, we also evaluate the impact of two different ways for categorising ethnicity: NER and ONS (1,2). NER consists of 9 different categories with higher granularity for 'Asian' and 'Black' groups while ONS consists of 5 categories, both are detailed in method section (15,25)."

2)Discussion
I have very few suggestions for improvement. Only suggestion is that the discussion could be shortened and structured differently:

1) First (quite long) paragraph of discussion is extensively on the completeness, yet this is not stressed in the abstract aims – if the authors want the completeness to be a main outcome, can they please include this in the methods / aims? As second paragraph then goes onto the ethnicity spine.

Thank you – we agree and have now covered this point more fully in the abstract and methods/aims to reflect this point.
"Outcome measures 1) Comparing the distribution of ethnicity records over time for four different selection approaches (Latest, Mode, Weighted-Mode and Composite) across age bands, sex, deprivation quintiles, health board, and residential location 2) distribution and completeness of records against the ONS census 2011."

"Results The distribution of the dominant group (White) is minimally affected based on the four different selection approaches. Across all other ethnic group categorisations, the Mixed group was most susceptible to variation in distribution depending on the selection approach used and varied from a 0.6% prevalence across the Latest and Mode approach to a 1.1% prevalence for the Weighted-Mode, compared to the 3.1% prevalence for the Composite approach. Substantial alignment was observed with the ONS 2011 census with the Latest group method (kappa= 0.68, 95% CI [0.67,0.71]) across all sub-groups. The record completeness rate was over 95% in 2021."

The details about data extraction are covered in the 'Study design and data sources' part of the method section. We have now expanded our detailing about quantifying the completeness of the records over time, which is covered in the 'Statistical Analysis' part of the method section. Reading as:

"Distribution of ethnicity records for each selection method (Latest, Mode, Weighted-Mode and Composite) and the two categorisation methods (NER and ONS) are compared over time. We also assessed record completeness over time. Three cohorts consisting of individuals alive and living in Wales in 2011, 2016 (mid-study) and 2021 (end-of-study) (Figure 1) were used to compare record completeness over time from the year the census was completed in 2011. Using the ONS and NER ethnic group categorisations, we compare the distribution of records for the four selection approaches across age bands, sex, deprivation quintiles, geographic health board and residential areas."

We further added this in the summary points reading as:
"Achieving ethnicity record completeness of over 95% in 2021."

2) Second paragraph-Again I think the terminology needs to be clear what categorisation and selection refer to, even if it is already clear in methods.

We agree and have reflected thoroughly on this point with added details earlier in the intro and method section. Details and direct quotes to where changes occurred are covered above in point 1.

3) The paragraph "Our study demonstrates that the Latest, Mode and Weighted-Mode approaches all provide suitable categorisations for ethnic group data and the choice of ethnic group methodological approach chosen by any study down to their respective study design, with the standard choice from current studies across the UK being the Latest (Figures 3 & 4 - Table 3)."

This paragraph seems rather out of place and disjointed from the rest of the discussion all on its own. Can the authors please improve the flow – prob better incorporated into a paragraph higher up?

Thank you, this has been moved to a higher paragraph as suggested.

4) Suggest to add in a more generic closing/conclusion statement in discussion:
"In conclusion, our development of the population-scale ethnicity spine provides robust ethnicity measures to healthcare research in Wales and a template which can easily be deployed in other TREs in the UK and beyond"

Thank you, we found your suggested statement a correct reflection of the work and have now updated this. The final conclusion of abstract reads as:
"In conclusion, our development of the population-scale ethnicity spine provides robust ethnicity measures for healthcare research in Wales and a template which can easily be deployed in other TREs in the UK and beyond."

5) there are a lot of supplementary files and it may be worthwhile to consider smaller font / tables

Thank you for this point – using your suggestion on font size, we have majorly reduced the length of supplement from 95 pages to 53 pages. Within the supplementary file we ensured all materials are accessible and within journal guidelines, and to support the transparency, reproducibility and transferability of the methodology and underlying code lists for readers of the paper. There is now a table of content directing readers to each one of the figures within the documents.

Minor

Typos
"Errornous" page 4 line 41/42 needs correcting please.

We have replaced this with a more exact term: "duplicated".

References

1. Khunti K, Routen A, Banerjee A, Pareek M. The need for improved collection and coding of ethnicity in health research. J Public Health (Bangkok) [Internet]. 2021 Jun 7 [cited 2022 Jun 10];43(2):e270–2. Available from: https://academic.oup.com/jpubhealth/article/43/2/e270/6024749

2. ONS. Ethnic group, national identity and religion - Office for National Statistics [Internet]. 2011 [cited 2022 Jun 10]. Available from: https://www.ons.gov.uk/methodology/classificationsandstandards/measuringequality/ethnicgroupnationalidentityandreligion
